# A novel trypsin of *Trichinella spiralis* mediates larval invasion of gut epithelium via binding to PAR2 and activating ERK1/2 pathway

**DOI:** 10.1371/journal.pntd.0011874

**Published:** 2024-01-02

**Authors:** Lu Lu Han, Qi Qi Lu, Wen Wen Zheng, Yang Li Li, Yan Yan Song, Xin Zhuo Zhang, Shao Rong Long, Ruo Dan Liu, Zhong Quan Wang, Jing Cui

**Affiliations:** Department of Parasitology, Medical College, Zhengzhou University, Zhengzhou, China; University of Liverpool, UNITED KINGDOM

## Abstract

**Background:**

Proteases secreted by *Trichinella spiralis* intestinal infective larvae (IIL) play an important role in larval invasion and pathogenesis. However, the mechanism through which proteases mediate larval invasion of intestinal epithelial cells (IECs) remains unclear. A novel *T*. *spiralis* trypsin (TsTryp) was identified in IIL excretory/secretory (ES) proteins. It was an early and highly expressed protease at IIL stage, and had the potential as an early diagnostic antigen. The aim of this study was to investigate the biological characteristics of this novel TsTryp, its role in larval invasion of gut epithelium, and the mechanisms involved.

**Methodology/Principal finding:**

TsTryp with C-terminal domain was cloned and expressed in *Escherichia coli* BL21 (DE3), and the rTsTryp had the enzymatic activity of natural trypsin, but it could not directly degrade gut tight junctions (TJs) proteins. qPCR and western blotting showed that TsTryp was highly expressed at the invasive IIL stage. Immunofluorescence assay (IFA), ELISA and Far Western blotting revealed that rTsTryp specifically bound to IECs, and confocal microscopy showed that the binding of rTsTryp with IECs was mainly localized in the cytomembrane. Co-immunoprecipitation (Co-IP) confirmed that rTsTryp bound to protease activated receptors 2 (PAR2) in Caco-2 cells. rTsTryp binding to PAR2 resulted in decreased expression levels of ZO-1 and occludin and increased paracellular permeability in Caco-2 monolayers by activating the extracellular regulated protein kinases 1/2 (ERK1/2) pathway. rTsTryp decreased TJs expression and increased epithelial permeability, which could be abrogated by the PAR2 antagonist AZ3451 and ERK1/2 inhibitor PD98059. rTsTryp facilitated larval invasion of IECs, and anti-rTsTryp antibodies inhibited invasion. Both inhibitors impeded larval invasion and alleviated intestinal inflammation *in vitro* and *in vivo*.

**Conclusions:**

TsTryp binding to PAR2 activated the ERK1/2 pathway, decreased the expression of gut TJs proteins, disrupted epithelial integrity and barrier function, and consequently mediated larval invasion of the gut mucosa. Therefore, rTsTryp could be regarded as a potential vaccine target for blocking *T*. *spiralis* invasion and infection.

## Author summary

*Trichinella spiralis* is an important foodborne parasitic nematode, and the intestinal infective larvae (IIL) invasion of host’s intestinal mucosa is the key step in *T*. *spiralis* infection. But the mechanism of the IIL invasion of gut epithelium has not been completely elucidated. Characterization of interaction between the IIL invasive proteins and their receptors in intestinal epithelium cells will be valuable to understand larval invasion mechanism, and contribute in development of preventive veterinary vaccines against *T*. *spiralis* infection. Our results indicated that a novel *T*. *spiralis* trypsin (TsTryp) binding to PAR2 in intestinal epithelium activated ERK1/2 pathway, decreased expression of gut tight junctions (TJs) proteins, disrupted gut epithelial integrity and barrier function, and consequently mediated larval invasion of gut mucosa. Therefore, TsTryp may be regarded as a potential vaccine target to block *T*. *spiralis* infection.

## Introduction

*Trichinella spiralis* is one of the widely distributed foodborne parasitic nematodes in the world [1]. Human *T*. *spiralis* infection is caused by eating raw or improperly cooked meat infected with infectious muscle larvae (ML) [2]. From 2009 to 2020, 8 human trichinellosis outbreaks consisting of 479 cases and 2 deaths occurred in China, and 7 (87.50%) of these outbreaks were a result of the ingestion of raw or semi-cooked pork [3]. *Trichinella* infection is not only a major public health issue but also a risk to meat safety. Nevertheless, it is difficult to eliminate *Trichinella* infection in animals because *Trichinella* has a diverse range of natural hosts and commercial veterinary anti-*Trichinella* vaccines are no available up to now [4]. Therefore, novel *Trichinella* invasion-related proteins with strong immunogenicity need to be identified, and preventive anti-*Trichinella* vaccines with high protection are necessary to be developed to eliminate *Trichinella* infection in food animals [5,6].

When infected meat is ingested, the encapsulated ML is released from the capsules in the stomach under the action of digestion fluids. The ML are exposed to bile and enteral contents in the small intestine and are activated into the intestinal infective larvae (IIL) [7,8]. The IIL penetrates the intestinal epithelium where it undergoes four molts within 31 h post infection (hpi) and develops into the adult worm (AW) stage [9]. Adult females mate with the males and give birth to the newborn larvae (NBL). The NBL enter the blood system and invade skeletal muscles, and are encapsulated to complete the lifecycle [10]. Gut epithelium is the first natural physical barrier against *Trichinella* intrusion and infection; it is also the prior interaction site between the IIL worms and the host [11,12]. However, the mechanism of IIL intrusion into the gut epithelium has not been completely elucidated. Characterization of the interaction between IIL invasive proteins and their receptors (or ligands) will be valuable for understanding the larval invasion mechanism, which would contribute to the development of preventive vaccines against *T*. *spiralis* infection [13,14].

The physical barrier of the gut mucosa, which is mainly composed of intestinal epithelial cells (IECs) and the junctional complex, is crucial for defense against the invasion of pathogens. The junctional complex is generally described as intercellular “palisades”, including tight junctions (TJs) complex, adherens junctions, and desmosomes [15]. The TJs are protein complex in intestinal epithelial barrier that regulate and control paracellular permeability. The physiological function of TJs is mainly maintained by three groups of proteins, including occludin, claudins and junction adhesion molecule (JAM) [16]. E-cadherin (E-cad) is a transmembrane glycoprotein that maintains the tight connection between cells and the integrity of its tissue structure, as well as the integrity of the intestinal barrier. Disruption of TJs and E-cad leads to intestinal barrier dysfunction, which is more conducive for intestinal helminthes such as *T*. *spiralis* to invade IECs [17]. The intestinal epithelial Caco-2 cell monolayers are often used in *in vitro* models to evaluate intestinal barrier function. We already showed that the serine proteases and cysteine proteases in excretory/secretory (ES) proteins of *T*. *spiralis* IIL degrade the TJs protein and mediate the IIL invasion of Caco-2 monolayers [18]. In addition, various *T*. *spiralis* serine proteases were identified in IIL proteins by immunoproteomics [11,19–20] and reported to participate in larval invasion of the gut epithelium [21–23]. However, the mechanism by which these *T*. *spiralis* proteases damage epithelial barrier integrity is not clear; namely, it is unknown whether these proteases directly hydrolyze or downregulate TJs protein, disrupt the gut epithelial barrier and mediate larval intrusion.

There are many enteral exogenous proteases in the gut, and the intestinal mucosa is often exposed to proteases secreted by intestinal parasites. In addition to digestion and degradation, these parasite-derived proteases can also act as relevant signaling molecules to regulate specific cell functions, such as activating protease-activated receptors (PARs) [24,25]. PARs are a unique family of G-protein-coupled receptors, including protease activated receptors 1 (PAR1), PAR2, PAR3, and PAR4 [26]. PARs are cell surface transmembrane proteins, PAR2 out of them is mainly expressed in IECs and immune cells throughout the gastrointestinal tract [27]. Activation of PAR2 in the gut has been reported to trigger cellular proliferation, chemotaxis, ion transport, and epithelial barrier function regulation. Although trypsin has been shown to be a potential agonist for PAR2 activation [28], it has been reported that the PAR2 is also involved in Th2 responses against *T*. *spiralis* infection [29]. However, the interaction between *T*. *spiralis* proteases and PAR2 receptors in the gut epithelium and its role in larval invasion has not been reported to date.

In our previous studies, a novel *T*. *spiralis* trypsin (TsTryp, GenBank: XM_003381619.1) was identified in IIL ES antigens by immunoproteomics with early infection sera, and the TsTryp gene was highly expressed in the invasive IIL stage [11,19–20,30]. TsTryp was mainly localized in the cuticle and stichosome of this nematode and it is a surface and secretory antigen. This study aimed to investigate the biological function of TsTryp in larval invasion of the gut mucosa and the molecular mechanisms involved. Furthermore, previous study indicated that trypsin cleaved PAR2 at the extracellular N-terminus to expose its tethered ligand domain, and then activated the PAR2 receptor and downstream pathway [27]. Activation of PAR2 caused intestinal inflammation and damaged the gut epithelial barrier. Therefore, we focused on the PAR2 as a target of TsTryp in the current study.

## Materials and methods

### Ethics statement

This study was performed in the light of National Guidelines for Experimental Animal Welfare (Minister of Science and Technology, People’s Republic of China, 2006). All experiments in this study were approved by the Life Science Ethics Committee of Zhengzhou University (No. ZZUIRB GZR 2021–0044).

### Parasites and animals

*Trichinella spiralis* isolate (ISS534) was collected from a naturally infected swine from central China [31]. The nematode was passaged in BALB/c mice, and the 4–6 weeks old female mice were obtained from the Experimental Animal Center of Zhengzhou University.

### Collection of IIL stage and ES antigens

IIL was collected from the intestines of the infected mice at 6 hpi. The IIL excretory/secretory (ES) antigens were prepared as described previously [32]. Briefly, after the IIL worms were thoroughly washed with sterile saline and serum-free RPMI 1640 medium (100 U penicillin/ml and 0.1 mg/ml streptomycin), they were cultured in 5 000 worms/ml medium 37°C and 5% CO_2_ for 18 h. The culture supernatant was concentrated using an Amicon Ultra-3 centrifugal filtration device (MW cut-off value:3 kDa) and centrifuged at 4°C, and 5000 × *g* for 1 h. IIL ES antigens were obtained and stored at −80°C until use [29].

### Preparation of rTsTryp and anti-rTsTryp antibody

TsTryp contains two similar trypsin-like domains, the N-terminal and C-terminal domains. In this study, we cloned and expressed the TsTryp C-terminal domain with a molecular weight of 26 kDa. Total RNAs was extracted from the IIL stage using TRIzol reagent (Invitrogen, USA). The coding sequence of the TsTryp C-terminal domain was amplified by PCR using specific primers containing the restriction sites for *Kpn*I and *Sal*I (**bold**). (F: 5′-AT**GGTACC**GGTGGATGGGAAACAAGACCCAATTC-3′; R: 5′-GC**GTCGAC**AGTTTCTTTCAACC AATCC-3′). The PCR products with 717 bp were cloned into the expression plasmid pQE-80L, and the recombinant expression plasmid pQE-80L/TsTryp was transferred into *Escherichia coli* BL21 (DE3) (Novagen, USA). Recombinant TsTryp (rTsTryp) were induced under the condition of 0.5 mM IPTG at 37°C for 6 h [33], rTsTryp was purified by Ni–NTA Sefinose resin (Sangon Biotech Co., Shanghai, China) [21].Then, it was re-natured by gradient dialysis for further study. Purified rTsTryp was analyzed by SDS-PAGE and Western blotting, as previously described [5,8].

Each of 20 mice was injected subcutaneously with 20 μg rTsTryp emulsified with complete Freund’s adjuvant, and boosted twice using 20 μg rTsTryp emulsified with incomplete Freund’s adjuvant at a 2-week-interval [7,14]. Two weeks after the third immunization, tail blood was collected and anti-rTsTryp immune sera were isolated, the antibody titer of anti-rTsTryp IgG was assayed by rTsTryp-ELISA as reported before [34].

### Assay of rTsTryp enzymatic activity

rTsTryp was re-natured according to the reported method [35], the refolded rTsTryp was dissolved in Buffer D (50 mM Tris-HCl, 100 mM NaCl, 10 mM CaCl_2,_ pH 7.5). Trypsin hydrolyzes the synthesized N-benzoyl-L-arginine ethyl ester (BAEE) to N-benzoyl-L-arginine (BA). Under trypsin catalysis, the ester bond increases with hydrolysis of BAEE, the hydrolysis product BA also increases gradually, and the ultraviolet (UV) absorption of the reaction system also increases accordingly. To determine the activity of rTsTryp, various rTsTryp concentrations (0.01, 0.02, 0.04, 0.06, 0.08 and 0.10 μg/μl) were added to the buffer with different pH values (pH 4.0–5.0 citrate buffer, pH 6.0–7.0 sodium phosphate buffer, pH 8.0–9.0 Tris–HCl buffer, pH 10.0–11.0 glycine buffer) and incubated at different temperature (20–70°C) for 40 min. BAEE (1 mM) was added and incubated at 25°C for 6 min, and the OD values at 253 nm were assayed by spectrophotometer every 30s [36]. Moreover, effect of metal ions (Ca^2+^, Ni^2+^, Cu^2+^, Zn^2+^, Co^2+^ and Mn^2+^) on rTsTryp enzyme activity was assessed at a concentration of 1.0 mM metal ions [37]. Different enzyme inhibitors, 5 μM E-64, 1 mM phenylmethylsulfonyl fluoride (PMSF), 10 μM ethylene diaminetet raacetic acid (EDTA), 1mM pepstatin A and 1mM 1,10-Phenanthroline (1,10 phe), were also used to further confirm the rTsTryp activity [13].

Furthermore, the proteolytic activity of rTsTryp was also determined by substrate gel electrophoresis containing 0.1% gelatin substrate [38]. The 12% substrate gel was loaded with the rTsTryp and run at 120 V for 150 min under the non-reducing conditions. After electrophoresis, the gels were washed twice with 2.5% Triton X-100 for 2 h to remove the SDS. The gels were cut according to the gel pores and incubated in 0.1 M phosphate buffer (pH 6 and 7) or 0.05 M Tris-HCl buffer (pH 8) with 0.05% NaN_3_ for 24 h at 37°C. Finally, the gels were stained with Coomassie brilliant blue and distained until transparent bands appeared [29].

### Cell culture and cell viability assay

The human colon adenocarcinoma cell line, Caco-2, was obtained from the Shanghai Institute for Biological Sciences of the Chinese Academy of Sciences. The mouse IECs were isolated from the small intestine of normal BALB/c mice in our laboratory [7]. Mouse striated muscle myoblasts (C2C12) were used as insensitive cells for *T*. *spiralis* invasion. Caco-2, IECs, and C2C12 were grown in Modified Eagle’s medium (MEM, Sigma Aldrich, USA) and Dulbecco’s modified Eagle’s medium (DMEM, Gibco, MA, USA), respectively; the media were supplemented 10% fetal bovine serum (FBS, Gibco), 0.1 mg/ml streptomycin, and 100 U/ml penicillin at 37°C with 5% CO_2_. Caco-2 cell medium was additionally supplemented with 1% non-essential amino acids (Solarbio, Beijing, China) [18]. The cells were passaged by conventional trypinization and exchanging medium every two days [33,39].

After the cells were digested with trypsin, these cells (5×10^4^ cells/well) were inoculated into a 96-well plate and cultured for 24 h in 5% CO_2_ at 37°C. Different concentrations of rTsTryp (0–25 μg/ml) were added to the wells and incubated for 3 h under the same conditions. After incubation, the medium was discarded, and 100 μl/well medium containing 10 μl CCK8 reagent (APTBIO; Shanghai, China) was added to each well of the 96 well plates. After incubation for 1 h, the absorbance at 450 nm was measured using a plate reader (Tecan, Switzerland) to evaluate the cellular viability [40].

### Immunofluorescence assay (IFA)

The binding of rTsTryp to IECs was investigated by IFA as reported previously [14,41]. The cells were grown on a glass coverslip in 6-well plates and maintained until confluence, and 20 μg/ml rTsTryp was added to cell monolayers and incubated for 2 h at 37°C and 5% CO_2_. After washing with cold PBS, the Caco-2 cell monolayers were fixed with 4% formaldehyde solution for 20 min and permeabilized with 0.1% Triton X-100 for 10 min at room temperature. After washing again with PBS, the monolayers were blocked with 10% goat serum and probed with 1:20 dilutions of infection serum, anti-rTsTryp serum, pre-immune serum, or anti-TRX serum. Goat anti-mouse IgG-Alexa Fluor 488 conjugate (1:100; Servicebio, Wuhan China) was used as the secondary antibody. After washing again, the cells were examined under fluorescence microscopy and confocal microscopy (Olympus, Japan) [4].

Furthermore, to assess the degradation of rTsTryp on TJs proteins, Caco-2 monolayers were incubated with rTsTryp. Specific antibodies against human ZO-1 (1:500) (Servicebio), occludin (1:160), claudin-1 (1:16) (Santa Cruz, USA), E-cad (1:500), and PAR2 (1:50) (Abcam, UK) were used as first antibodies. After washing with PBS, Caco-2 cells were incubated with species-specific Alexa Fluor 488- or CY3-labeled antibodies (1:100) (Servicebio) at 37°C for 2 h, the cell nuclei were stained with DAPI, and the cell monolayer was observed under fluorescence microscopy (Olympus, Japan) [18].

### ELISA determination of binding of rTsTryp to IECs

The ability of rTsTryp to bind to IECs was determined by ELISA [20]. IEC cells were digested by trypsin and collected by centrifugation at 300 *g* for 5 min. IEC soluble proteins were prepared through cell lysis and sonication on ice, the concentration of IECs soluble protein was determined as reported before [42]. A 96-well plate (Corning, USA) was coated with IEC proteins at different concentrations (0.125, 0.25, 0.5, 0.75, 1, 1.25, 1.5, and 2 μg/ml) overnight at 4°C, and the plate was blocked with 5% skim milk in PBST at 37°C for 1 h. After washing with PBST, the plate was incubated with 2 μg/ml rTsTryp at 37°C for 2 h, and then probed with 1:100 dilutions of anti-rTsTryp serum, anti-TRX tag serum, infection serum, and normal mouse serum at 37°C for 1 h. After washes with PBST again, the plate was incubated with horseradish peroxidase (HRP)-conjugated anti-mouse IgG (1:10 000, Southern Biotech., USA). Finally, the absorbance at 492 nm was measured after color development and termination [12,13]. After the optimal IEC protein coating concentration (1.5 μg/ml) was ascertained, the plate was coated with 1.5 μg/ml IEC protein, and incubated with different concentrations of rTsTryp (0.25, 0.5. 0.75, 1, 1.25, 1.5, 1.75, and 2 μg/ml). The subsequent procedures were the same as above-mentioned.

### Binding of rTsTryp to IEC assessed by Far-Western blot

The lysate supernatant of IECs cells incubated with rTsTryp was analyzed by 12% SDS-PAGE to evaluate the interaction between rTsTryp and IECs, C2C12 cell proteins was served as a negative control [19]. After electrophoresis, the proteins were transferred to nitrocellulose (NC) membranes (Millipore, USA). The membranes were blocked with 5% skim milk and incubated with rTsTryp at 37°C for 2 h. After washing with TBST, the membranes were cut into strips and probed with anti-rTsTryp serum, infection serum and pre-immune serum (1:100 dilutions) at 37°C for 1 h. The trips were incubated with HRP-labeled goat anti-mouse IgG conjugates (1:10 000; Southern Biotech) for 1 h at 37°C. After washing with TBST, the color development of the strips was performed with 3, 3’-diaminobenzidine tetrahydrochloride (DAB; Sigma), and terminated by washing the strips with deionized water [5]. Finally, the bands in the strips were analyzed using AlphaView software [43].

### qPCR

To evaluate the effects of rTsTryp on the transcription levels of gut epithelial TJs proteins (ZO-1, occludin, claudin-1, and E-cad), Caco-2 monolayers were pre-incubated with 20 μg/ml rTsTryp at 37°C for 2 h. Total RNA was extracted from Caco-2 cells pretreated with rTsTryp using TRIzol reagent (Invitrogen, USA). The RNA concentration was detected, and quality was assessed using a NanoDrop 2000 (Thermo Fisher, USA) and then detected by electrophoresis. TJs protein cDNA was synthesized according to the manufacturer’s instructions (TaKaRa, Japan). qPCR was performed using the Applied Biosystems 7500 Fast System (Life Technologies, USA) [44,45]. Specific primers for ZO-1, E-cad, occludin, claudin-1, and glyceraldehyde 3-phosphate dehydrogenase (GAPDH) were synthesized by Sangon Biotech (Shanghai, China) (S1 Table). Relative mRNA expression of TJ genes was normalized using GAPDH as an internal control. Gene expression results were calculated using the 2^−ΔΔCt^ method [9,13]. Each sample was tested in triplicate. Moreover, the transcription levels of murine gut TJs proteins and cytokines (TNF-α, IL-1β, IL-4, and IL-10) were also assessed by qPCR after inhibitors were used and challenged with ML in mice.

### Western blot analysis of expression of TJs in rTsTryp-treated Caco-2 monolayer

To investigate the rTsTryp degrading or down-regulating expression of gut epithelium TJs proteins and related pathways, soluble proteins from Caco-2 monolayers pretreated with 20 μg/ml rTsTryp at 37°C for 2 h were analyzed by western blotting as described before [14,37]. Briefly, Caco-2 cell monolayers were incubated with rTsTryp at 37°C for 2 h, cell proteins were collected, and the cell lysates were separated by 10% SDS-PAGE and transferred subsequently onto PVDF membrane (Millipore, USA). The membrane was blocked with 5% skimmed milk in TBST for 1 h at 37°C, and cut into strips. Subsequently, the strips were probed overnight at 4°C with antibodies against ZO-1 (1:1 000, Servicebio, Wuhan, China), E-cad (1:200, Santa Cruz, USA), occludin (2 μg/ml, Santa Cruz), claudin-1 (2 μg/ml, Santa Cruz), PAR2 (1:1 000, Abcam, UK), p-ERK1/2 (1:1 000, Abmart, Shanghai), ERK1/2 (1:1 000, Abmart, Shanghai), GAPDH (1:1 000), and β-actin (1:2 000) (Servicebio). After washing with TBST, the strips were incubated with HRP -conjugated anti-mouse IgG or anti-rabbit IgG as secondary antibodies (1:10 000). Finally, the color of the strips was developed using an enhanced chemiluminescence kit (CWBIO, Beijing, China). The results for the above proteins were normalized using GAPDH as a loading control [4,18].

### Western blotting of rTsTryp directly degrading TJs proteins in Caco-2 cells

To further verify whether rTsTryp damaging gut epithelial integrity contributes to direct hydrolysis or decreases expression of TJs proteins in Caco-2 monolayers, the *in vitro* direct hydrolysis of TJs proteins by rTsTryp was conducted as described previously [46,47]. Soluble Caco-2 cell proteins (200 μg) were incubated with 5 μg rTsTryp at pH 8.5, 37°C for 12 h; 5 μg trypsin was served as a positive control, while denatured rTsTryp at 100°C for 5 min was used as a negative control. The content of TJs proteins in Caco-2 cells was analyzed by SDS-PAGE and western blotting [37].

### Co-immunoprecipitation (Co-IP)

Co-IP is based on the specific binding of Protein A/G agarose to IgG antibodies. To investigate whether rTsTryp binds to PAR2 in Caco-2 cells, Co-IP was conducted as previously reported [48, 49]. Briefly, Caco-2 cells were cultivated until they reached complete confluence. The Caco-2 monolayer was pre-incubated with 20 μg/ml rTsTryp for 2 h. After washing with PBS, the monolayers were lysed in DISC buffer [1% Triton X-100, 30 mM and pH 7.0 Tris–HCl, 10% glycerol, 120 mM NaCl, 1 mM PMSF and 1 mM complete protease inhibitor cocktail (Sangon Biotech)] and incubated on ice for 1 h. The lysate supernatant was collected by centrifugation at 10 000 *g* at 4°C for 10 min and reacted with protein A/G agarose beads for 2 h at 4°C. The supernatant was collected by centrifugation at 400 **×** g for 5 min. The supernatant with non-specific binding proteins removed was incubated with anti-rTsTryp antibody (normal mouse IgG as control) and new Protein A/G beads for 6 h at 4°C. After incubation, the supernatant was discarded, and the beads were collected and washed with DISC buffer. Finally, the bound proteins (protein A/G-rTsTryp-PAR2) were eluted from the beads, denatured by boiling for 5 min, and then separated by 12% SDS-PAGE and analyzed by western blotting, in which anti-rTsTryp antibody and anti-PAR2 antibody (1:1000; Abcam, UK) were used as the primary antibodies. The secondary antibody without light and heavy chains (1:5000, Abmart) was used to avoid IgG light and heavy chain pollution [50,51].

### Determination of paracellular permeability

When small molecules permeate the cell monolayer, the function of TJs proteins is damaged and paracellular permeability is increased. To ascertain the effect of rTsTryp disruption on the integrity of gut epithelial barrier, dextran was used to test paracellular permeability of the Caco-2 monolayer. Culture medium containing rTsTryp was added to the upper compartment of a transwell plate covered with Caco-2 cells and incubated at 37°C for 2 h. After washing with PBS, the media containing 0.5 mg/ml FITC-dextran with 4 kDa (FD4, Sigma, USA) was added to the upper compartment of the transwell plate, while 1.3 ml of basal medium was added to the basolateral chamber of the plate [40,52]. The plate was slowly shaken in the dark at 37°C for 120 min. Then, 0.2 ml of liquid was removed from the base side chamber every 20 min and added to a black 96-well plate (Corning, USA) for fluorescence determination [53]. The fluorescence signal at 485 nm excitation wavelength and 520 nm emission wavelength was detected using a microplate reader (Tecan, Schweiz, Switzerland) [54]. The standard curve was calculated according to the change in absorbance of FD4 concentration from 0.025–1.6 μg/ml.

### Determination of PAR2 receptor and ERK1/2 pathway in Caco-2 cells by using rTsTryp and inhibitor

The activation of the basolateral PAR2 receptor in IECs promotes the activation of extracellular signal regulated kinase 1/2 (ERK1/2), which participates in the activation of F-actin and redistribution of ZO-1 on the cell membrane; thus, the ERK1/2 pathway plays a role in the regulation of tight junction proteins [55,56]. To verify whether rTsTryp binding to PAR2 disrupts TJ integrity by activating the ERK1/2 pathway and reduces the expression of TJ proteins, Caco-2 monolayer was pretreated with 10 μM AZ3451 (PAR2 antagonist; MCE, USA) at 37°C for 12 h, then incubated with 20 μg/ml rTsTryp and trypsin, and 1 μM 2-Furoyl-LIGRLO amide (2fAP, PAR2 agonist; MCE) for 2 h at 37°C. The ERK1/2 pathway inhibitor PD98059 (10 μM; MCE) were also used in this study based on the previous report [56]. After washing with PBS, soluble cell proteins were prepared, and western blotting was performed as previously reported [18,57]. Anti-PAR2 antibody (1:1 000; Abcam), rabbit anti-human p-ERK1/2 (1:1 000; Abmart, Shanghai, China), or rabbit anti-human ERK1/2 antibody (1/1 000, Abmart) were used to detect the expression of PAR2 and ERK1/2 pathway proteins in Caco-2 monolayers.

### The *in vitro* larval invasion test

To investigate whether rTsTryp promotes *T*. *spiralis* invasion of gut epithelium, an *in vitro* larval invasion test was carried out as described previously [7]. Briefly, the ML were activated to the IIL by 5% mouse bile for 2 h at 37°C, and 100 IIL were mixed with semi-solid medium containing various doses of rTsTryp (0–20 μg/ml), or different dilutions (1:100–1:1 600) of anti-rTsTryp serum, infection serum, normal serum or anti-TRX serum. This mixture was added to the Caco-2 monolayer and incubated for 2 h at 37°C. The larvae invading the monolayer were then observed and numbered under a microscope. Furthermore, to evaluate the role of the PAR2 receptor and ERK1/2 pathway, the Caco-2 monolayer was pretreated with 1 μM PAR2 agonists (2fAP), 10 μM PAR2 antagonist (AZ3451), 10 μM ERK1/2 inhibitors (PD98059), and AZ3451+PD98059, and then the medium containing the IIL was added to observe the IIL invasion of the cell monolayer. The invaded larvae had a snake-like action and migrated within the Caco-2 monolayer, whereas non-invaded larvae were spirally coiled on the surface of the Caco-2 monolayer [21,58]. rTsTryp and 2fAP promotion and inhibition of anti-rTsTryp antibodies, AZ3451 and PD98059 on larval invasion was calculated in comparison with its control group [59].

### Pretreatment of mice with inhibitors and larval challenge

One hundred and twenty-five mice were randomly divided into five groups (25 animals each): (1) Saline group: each mouse was intraperitoneally injected with 200 μl physiological saline; (2) solvent PEG300+DMSO group: each mouse was intraperitoneally injected with 100 μl 50% PEG300 and 100 μl 1‰ DMSO; (3) PAR2 antagonist AZ3451 group: each mouse was intraperitoneally injected with 100 μl AZ3451 solution and 100 μl 50% PEG300; (4) ERK1/2 pathway inhibitor PD98059 group: each mouse was intraperitoneally injected with 100 μl PD98059 solution and 100 μl 1‰ DMSO; (5) AZ3451+PD98059 group: each mouse was intraperitoneally injected with 100 μl AZ3451 solution and 100 μl PD98059 solution. For intraperitoneal injection, 50 μg/ml AZ3451 was dissolved in 1‰ DMSO, and PD98059 was administered at a dose of 10 mg/kg [60]. The inhibitors were administered to all mice three times (once a day on an alternate day for 5 days), and at 12 h after the inhibitors were injected, all mice were challenged orally with 200 *T*. *spiralis* ML.

Ten mice from each group were euthanized at 12 hpi, and enteral IIL was collected and numbered from infected mice as described previously [45]. The remaining 15 mice in each group were sacrificed at 5 dpi, intestinal adults were recovered from ten infected mice, and adult burden reduction was assessed based on the mean number of intestinal adults in the inhibitor group relative to those from the saline group [7].

At 5 dpi, intestinal tissues of the other five infected mice were obtained, total RNAs were isolated from murine intestines, and the mRNA expression levels of the TJs (ZO-1, E-cad, occludin and claudin-1) and cytokines (TNF-α, IL-1β, IL-4, and IL-10) were determined by qPCR. Soluble proteins of infected mouse intestines were also prepared, the expression levels of the PAR2 and TJs proteins in gut epithelium were assessed by using Western blotting [9]. Additionally, intestines from infected mice were fixed in 4% formalin for 24 h and embedded in paraffin wax; 3-μm-thick tissue sections were prepared, deparaffinized, and stained by immunohistochemistry (IHC) as reported before [61]. Briefly, the sections were treated with 0.01 M citrate buffer at 100°C for 5 min for antigen retrieval, followed by blocking endogenous peroxidase activity with 0.3% H_2_O_2_. After washing with PBS, the sections were blocked with 3% bovine serum albumin (BSA) at 37° C for 1 h. Then, the samples were incubated at 4°C overnight with anti-p-ERK1/2 antibody as primary antibody (1:200, Abmart). Following incubation with HRP-labeled goat anti-mouse IgG conjugates (1:10 000, Southern Biotech), intestinal sections were stained with diaminobenzidine (DAB; Sangon Biotech, Shanghai), redyed with hematoxylin, and examined under a microscope. Protein expression was analyzed using ImageJ pro-plus [14]. The protocol of inhibitor administration and *T*. *spiralis* infection is shown in S1 Fig.

### Intestinal permeability assay

Intestinal permeability was assessed by measuring the amount of 4 kDa FITC-dextran (FD 4) in the infected mouse blood plasma [25,62]. Five mice from each group were pretreated with AZ3451, PD98059, or AZ3451+PD98059, and then challenged with 200 *T*. *spiralis* ML. At 5 dpi, all mice were fasted overnight and 100 μl FD 4 was used to each mouse at a concentration of 50 mg/ml by intragastric administration. The mice then resumed drinking water. Four hours later, mouse blood was collected to separate the plasma and was kept away from light. The plasma was diluted 1:100 with PBS to measure the absorbance at 485 nm excitation wavelength and 520 nm emission wavelengths using a microplate reader [63]. The FD 4 was continuously diluted with PBS to make a standard curve.

### Datum presentation and statistical analysis

Data in this study are shown as arithmetic mean ± standard deviation (SD) and were statistically analyzed using SPSS software (version 22.0). The transcription and protein expression levels of PAR2, TJs proteins, and cytokines were analyzed using one-way ANOVA. The Chi-square test was used to analyze larval invasion between the two groups and among various groups. *P* < 0.05 was regarded as statistically significant.

## Results

### Western blot analysis of purified rTsTryp

**Fig 1:**
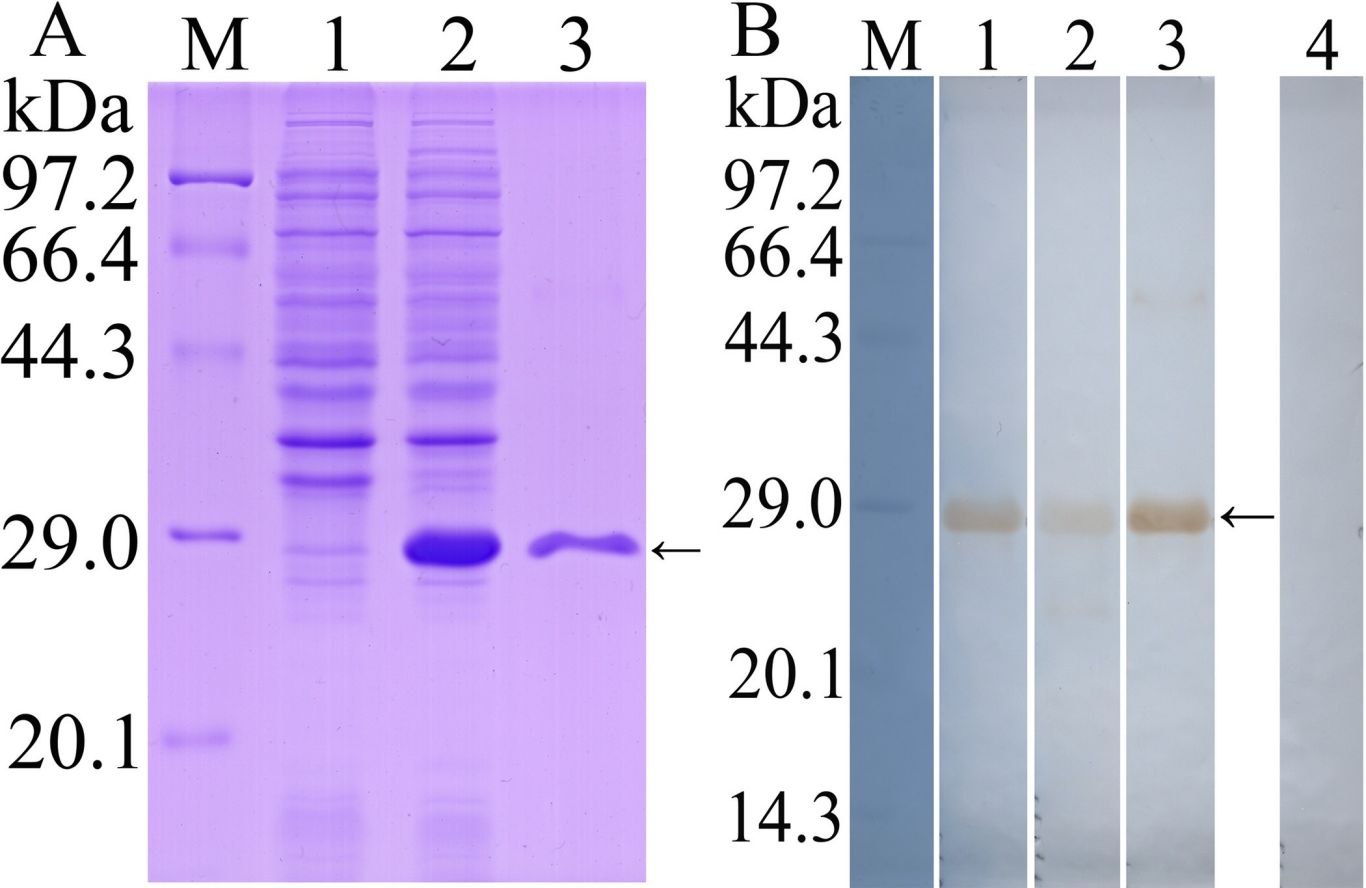
Western blot analysis of rTsTryp. **A:** SDS-PAGE analysis of rTsTryp. Lane M: protein marker; Lane 1: lysate of non-induced BL21 protein containing pQE-80L/TsTryp; Lane 2: lysate of induced BL21 protein containing pQE-80L/TsTryp; Lane 3: purified rTsTryp. **B:** Western blot analysis of rTsTryp. M: protein marker; Lane 1–4: rTsTryp was recognized by anti-His-tag antibodies (lane 1), *T*. *spiralis*-infected murine serum (lane 2) and anti-rTsTryp serum (lane 3), but not recognized by normal serum (lane 4). The arrow indicates rTsTryp with 26 kDa.

SDS-PAGE results showed that rTsTryp was expressed in precipitation, and the molecular weight (MW) of rTsTryp with the C-terminal domain purified by Ni–NTA Sefinose resin was 26 kDa, which was consistent with the predicted MW of rTsTryp (Fig 1A). Anti-rTsTryp serum was assayed by rTsTryp-ELISA, and the IgG antibody titer was 1:10^5^. Western blotting results revealed that rTsTryp was recognized by anti-His-tag antibodies, *T*. *spiralis*-infected murine serum, and anti-rTsTryp serum, but not by normal serum (Fig 1B).

### Enzymatic activity of rTsTryp for hydrolyzing the substrate

**Fig 2:**
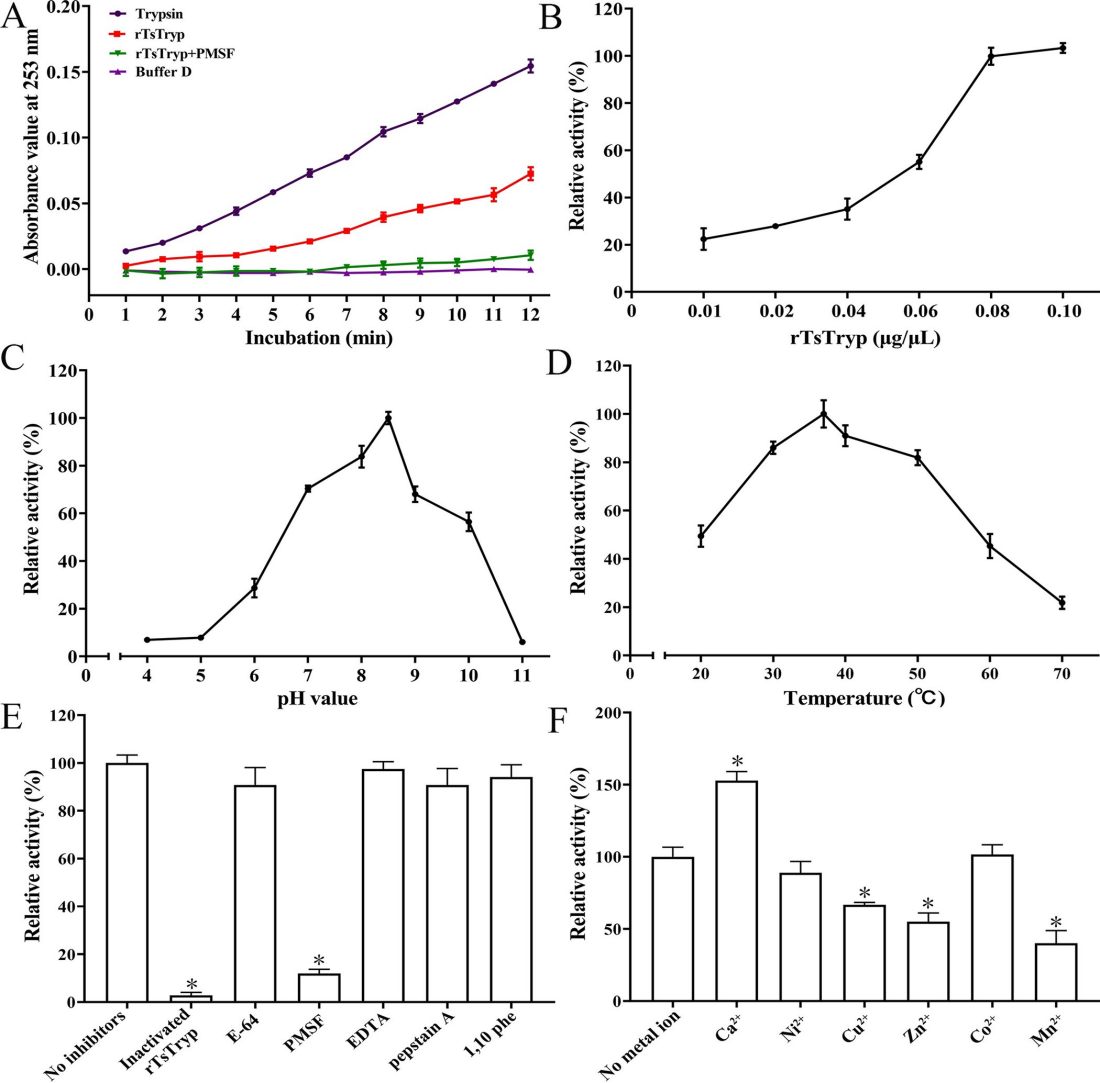
Enzyme activity assay of rTsTryp. **A:** N-benzoyl-l-arginine ethyl ester (BAEE) was hydrolyzed by rTsTryp; the trypsin, rTsTryp + PMSF and Buffer D were used as the controls. **B:** Enzyme activities of rTsTryp at different concentrations. **C:** Enzyme activities of rTsTryp at different pH values. **D:** Effect of different temperature on rTsTryp enzyme activity. **E:** Effect of different inhibitors on rTsTryp activity. **F:** Effect of different metal ions on rTsTryp enzyme activity, * *P* < 0.05 compared with blank controls without inhibitor or metal ion.

As shown in Fig 2A, rTsTryp hydrolyzed the substrate BAEE, similar to trypsin. However, Buffer D and rTsTryp + PMSF (a serine protease inhibitor) did not hydrolyze BAEE. The substrate hydrolysis rate increased with increasing rTsTryp concentration, and the optimum concentration of rTsTryp for hydrolyzing BAEE was 0.08 μg/μl (Fig 2B). The optimum pH and temperature of rTsTryp were 8.5 and 37°C, respectively (Fig 2C and 2D). The rTsTryp activity was significantly inhibited by PMSF, and the relative inhibition rate of PMSF on rTsTryp activity was 88.06% compared to the blank control without inhibitors (*F* = 1647.678, *P* < 0.0001) (Fig 2E). Among various metal ions used in this study, only the Ca^2+^ significantly increased rTsTryp activity, with a 52.78% increase in relative enzyme activity (*F* = 99.178, *P* < 0.01). Cu^2+^, Zn^2+^ and Mn^2+^ inhibited the rTsTryp enzyme activity with inhibition of 33.33% (*F* = 70.591, *P* < 0.01), 45% (*F* = 75.412, *P* < 0.01), and 60% (*F* = 88.363, *P* < 0.01), respectively (Fig 2F).

**Fig 3:**
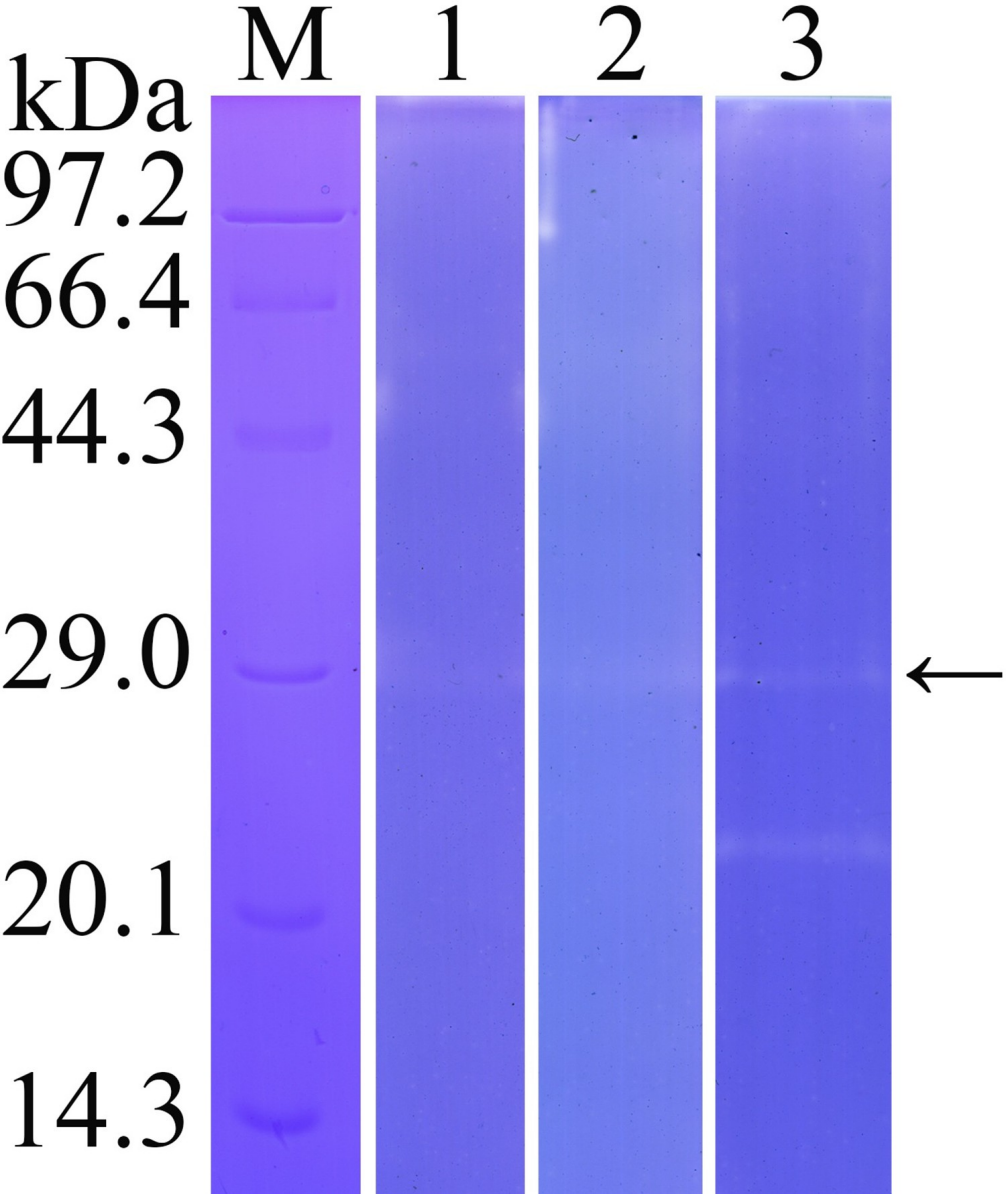
The effect of different pH on enzyme activity of rTsTryp was analyzed by gel zymography. Lane M: protein marker; Lane 1–3: pH 6, 7 and 8. The arrow indicates the 26 kDa band hydrolyzed by rTsTryp.

Zymography analysis showed that there was an obvious hydrolytic band with 26 kDa, indicating that rTsTryp had strong hydrolytic activity at pH 8. A clear hydrolysis band with 22 kDa could also be observed, suggesting that it may be a different folding form of rTsTryp (Fig 3).

### Effect of rTsTryp on viability of IECs and Caco-2 cells

The results of the CCK-8 assay showed that when the IECs and Caco-2 cells were treated with rTsTryp (0, 15, 20, and 25 μg/ml) for 3 h, cell viability was not evidently changed in comparison with the untreated control group (*F*_IECs_ = 0.406, *P* > 0.05; *F*_Caco-2_ = 0.396, *P* > 0.05) (S2 Fig). Therefore, 20 μg/ml rTsTryp was used in subsequent experiments.

### Binding of rTsTryp to IECs assayed by IFA

**Fig 4:**
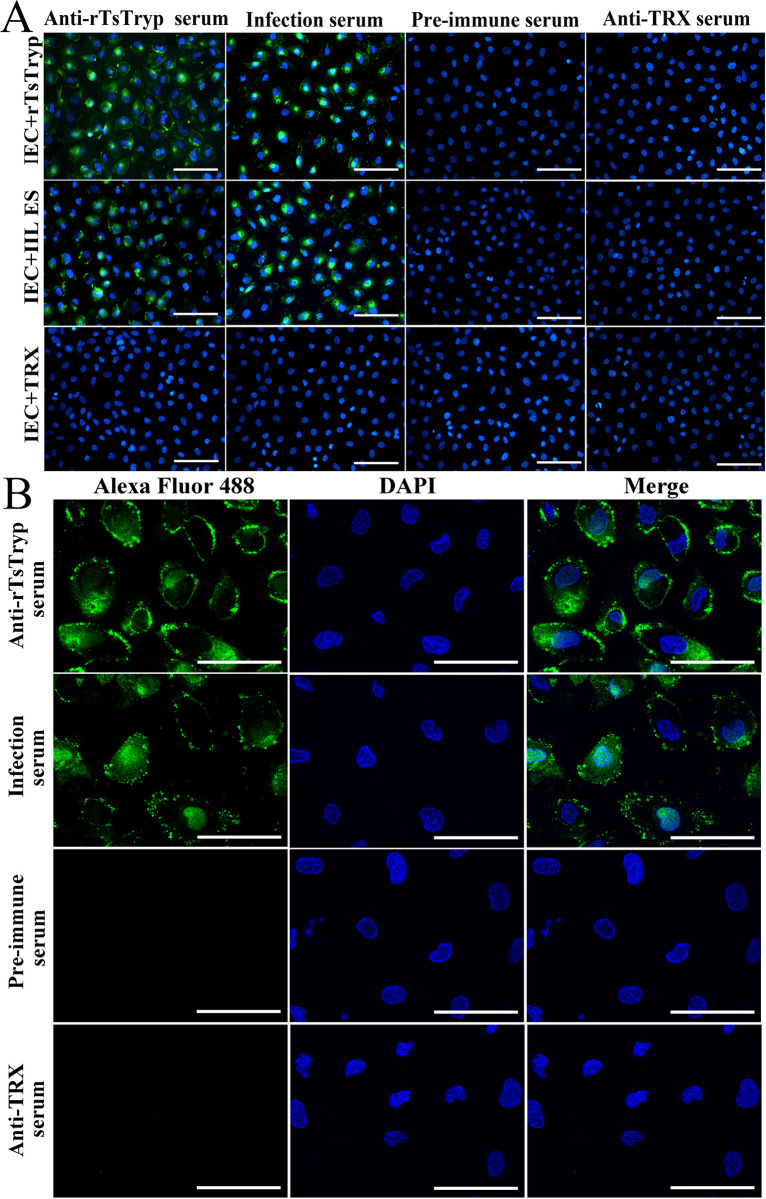
Binding of rTsTryp to IEC and its cellular localization by IFA. **A:** Binding of rTsTryp and IECs was observed by fluorescence microscope (400**×)**. **B:** Confocal microscopy showed that bind of rTsTryp and IEC was mainly located in cytomembrane and a little in cytoplasm. In the IFA test, the primary antibodies were *T*. *spiralis*-infected serum, anti-rTsTryp serum, normal serum and anti-TRX serum, respectively. Alexa Fluor 488-conjugated anti-mouse IgG was served as the secondary antibody. Nuclei were stained blue using DAPI. Scale bars: 5 μm

The IFA results showed that green fluorescence in IEC was detected by anti-rTsTryp serum and infected serum after co-incubation with rTsTryp. Confocal microscopy revealed that immunofluorescence staining was mainly located on the surface and cytoplasm of IECs. However, no fluorescent staining was detected using pre-immune serum and anti-TRX serum (Fig 4). The results demonstrated that there was a specific binding between rTsTryp and IECs, and the binding was mainly localized in the cell membrane and a little in cytoplasm of the IECs.

### Binding of rTsTryp and IEC proteins assayed by ELISA

The ELISA results showed that rTsTryp bound and interacted with IEC proteins. The OD value of ELISA was related to the concentration of IEC proteins (*r* = 0.980, *P* < 0.0001), and increased with increasing IEC protein concentration (*F* = 319.314, *P* < 0.0001). The binding capacity was also dose-dependent for rTsTryp (*r* = 0.987, *P* < 0.0001), and increased with increasing rTsTryp concentration (*F* = 519.754, *P* < 0.0001) (S3 Fig).

### Binding of rTsTryp to IEC assayed by Far-Western blot

SDS-PAGE results showed that the IEC soluble proteins had 25 bands (16.3–95.7 kDa). The results of Far-Western blot showed that after IEC proteins were incubated with rTsTryp, 20 protein bands of 15.0–91.6 kDa were identified by anti-rTsTryp serum, and 19 bands of 18.0–91.6 kDa were identified by infection serum. However, IEC proteins incubated with TRX tag were not recognized by anti-rTsTryp serum; pre-immune serum did not recognize any bands in IEC proteins incubated with rTsTryp (S4 Fig). Moreover, C2C12 cell proteins incubated with rTsTryp were not recognized by anti-rTsTryp serum or infection serum. The results demonstrated that there is a specific binding and interaction between rTsTryp and IEC proteins.

### rTsTryp disrupted TJs protein of Caco-2 cell monolayer

**Fig 5:**
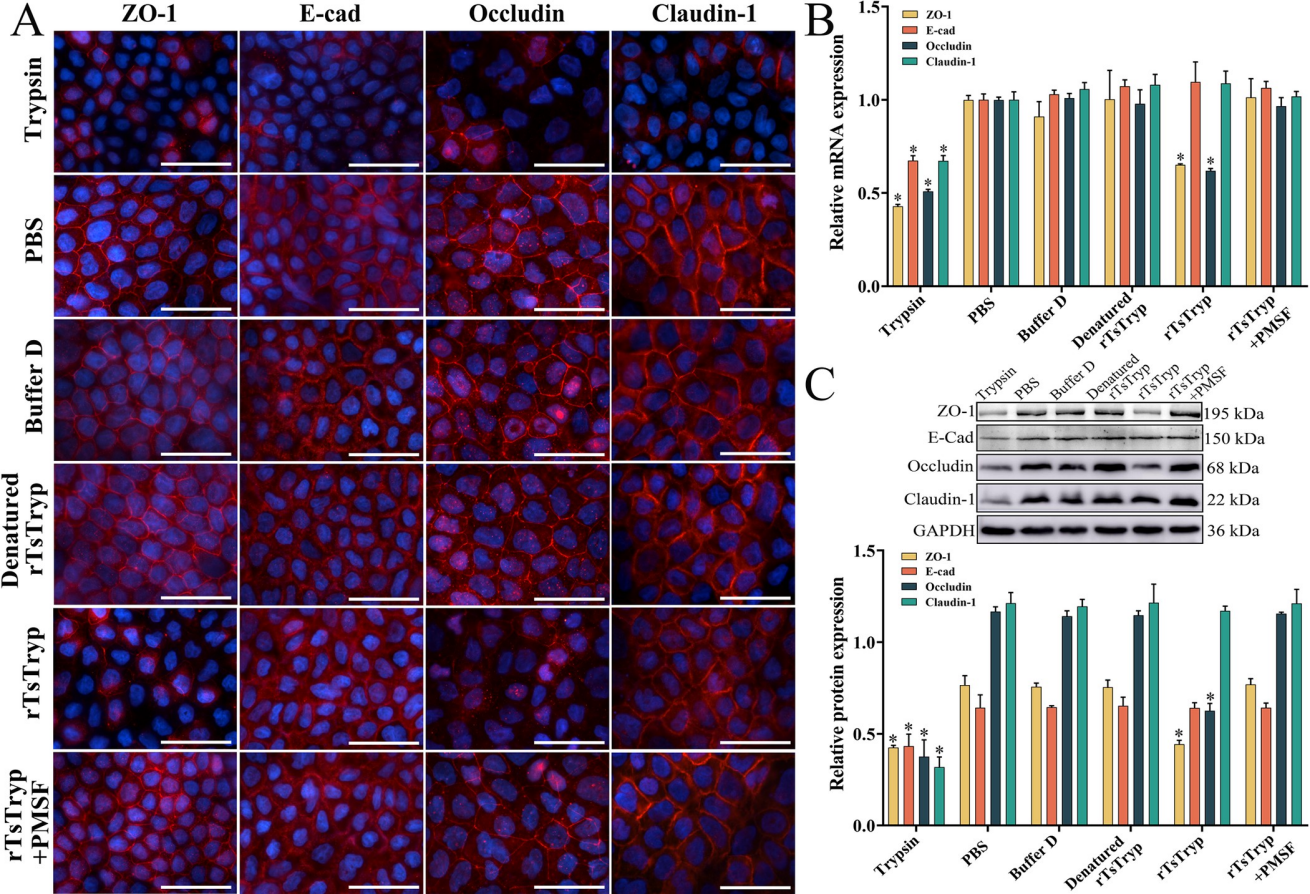
rTsTryp degrading or down-regulating TJ proteins in Caco-2 cell monolayer. **A:** IFA analysis of TJ proteins in Caco-2 monolayer treated with rTsTryp. Both ZO-1 and occludin were significantly down-regulated on Caco-2 monolayer incubated with rTsTryp, as demonstrated that the continuous immunostaining at cell-cell junction was obviously reduced or disappeared. Denatured rTsTryp represents the heating inactivated rTsTryp at 100°C for 5 min. **B**: qPCR analysis of rTsTryp effect on transcription level of TJs protein genes in Caco-2 monolayer. **C:** Western blotting analysis of the rTsTryp effect on expression level of TJ proteins in Caco-2 monolayer. Caco-2 cell monolayer was incubated with 20 μg/ml rTsTryp for 2 h, and GAPDH as a loading control. Expression levels of ZO-1, E-cad, occludin and claudin-1 relative to GAPDH were analyzed by gray-level analysis. Each group had triplicate. * *P* < 0.01 compared with PBS group. Scale bars: 5 μm.

To investigate whether rTsTryp degrades or down-regulates TJs proteins, Caco-2 cell monolayers were incubated with rTsTryp. IFA results showed that both the expression of ZO-1 and occludin were significantly down-regulated in Caco-2 cell monolayers incubated with rTsTryp, as demonstrated that the continuous immunostaining at cell-cell junctions was obviously reduced or disappeared compared to the PBS group. However, in Caco-2 cells incubated with denatured rTsTryp and rTsTryp+PMSF, the expression of ZO-1 and occludin showed no evident changes. Moreover, rTsTryp treatment had no obvious effect on claudin-1 and E-cad expression (Fig 5A).

qPCR results confirmed that the transcription levels of ZO-1 and occludin genes in Caco-2 cells treated with rTsTryp were significantly lower than those in the PBS group. The transcription levels of ZO-1 and occludin were reduced by 34.50 and 38.11% compared to PBS group (*F*_ZO-1_ = 626.155, *F*_occludin_ = 993.180, *P* < 0.0001). However, rTsTryp pretreatment did not have obvious effects on the transcription levels of the claudin-1 and E-cad genes (Fig 5B). Western blotting results further confirmed that when Caco-2 monolayers were incubated with 20 μg/ml rTsTryp for 2 h, the expression levels of ZO-1 and occludin was decreased by 42.09 and 46.34%, respectively, compared to the PBS group (*F*_ZO-1_ = 100.521, *P* < 0.01; *F*_occludin_ = 396.97, *P* < 0.0001) (Fig 5C). However, the expression levels of claudin-1 and E-cad in Caco-2 monolayers treated with rTsTryp did not have obvious change relative to the PBS group (*P* ˃ 0.05). Moreover, after Caco-2 monolayers were incubated with inactivated rTsTryp and rTsTryp+PMSF, the expression levels of ZO-1 and occludin were not significantly changed compared to those in the PBS group (*P* ˃ 0.05). The results suggested that rTsTryp-mediated degradation or down-regulation of ZO-1 and occludin expression is likely related to the enzymatic activity of rTsTryp.

### rTsTryp did not directly degrade TJs proteins in Caco-2 cells

**Fig 6:**
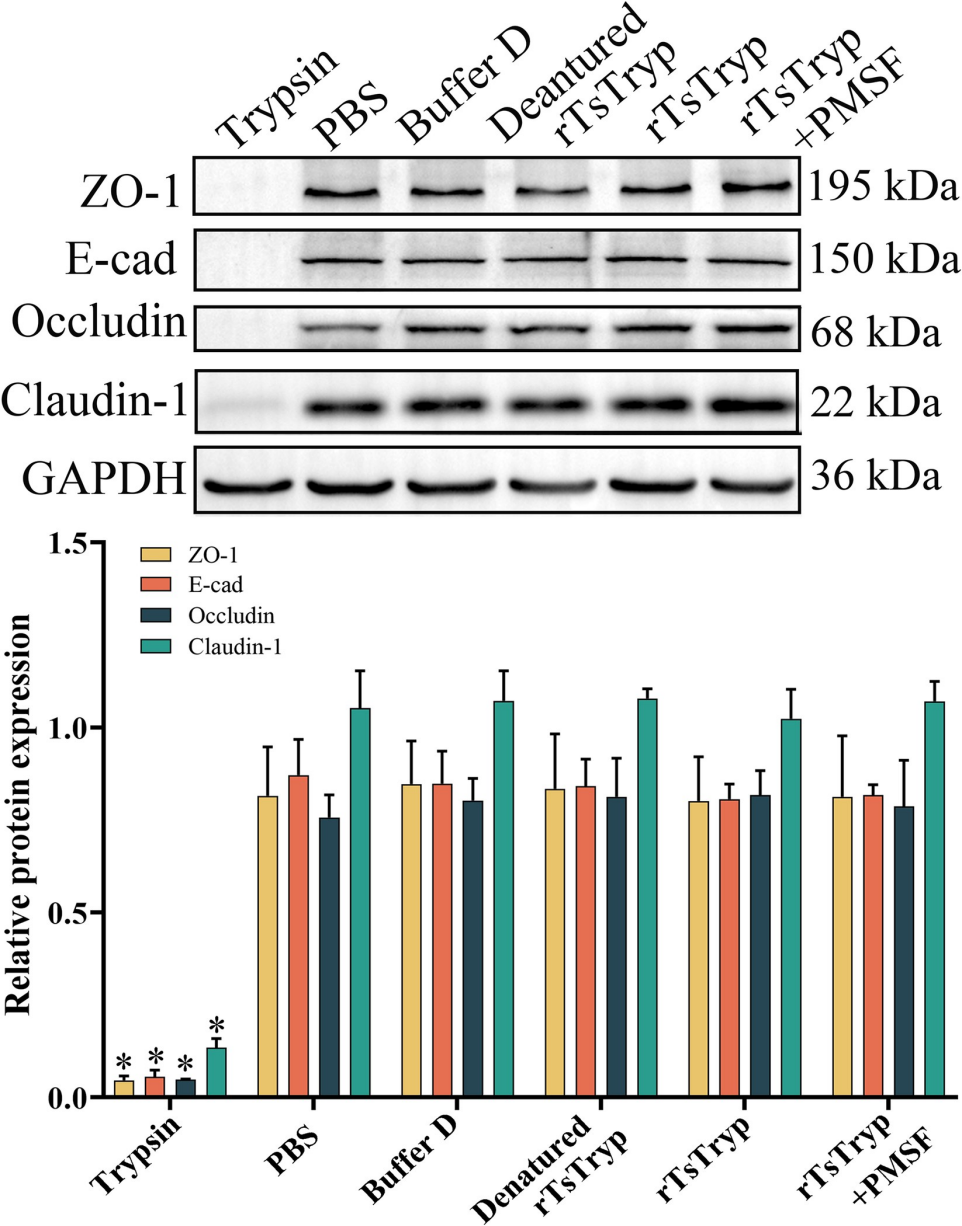
rTsTryp could not directly degrade TJs proteins in Caco-2 monolayer. Caco-2 cell soluble proteins (200 μg) was incubated with 5 μg rTsTryp at 37°C for 12 h, and GAPDH as a loading control. Expression levels of ZO-1, E-cad, occludin and claudin-1 relative to GAPDH were analyzed by gray-level analysis. Each group had triplicate. *P < 0.01 relative to the PBS group.

After soluble Caco-2 cell proteins were incubated with rTsTryp for 12 h, western blotting showed that rTsTryp did not directly degrade TJs proteins in the Caco-2 monolayers (Fig 6). Quantification showed that the contents of ZO-1, E-cad, occludin, and claudin-1 in rTsTryp-incubated Caco-2 cells were not evidently reduced relative to the PBS group (*P* > 0.05). However, when Caco-2 cell proteins were incubated with trypsin, the levels of ZO-1, E-cadherin, occludin, and claudin-1 were significantly reduced (*F*_ZO-1_ = 101.093, *P* < 0.01; *F*_E-cad_ = 206.776, *F*_occludin_ = 407.524, *F*_claudin-1_ = 234.365, *P* < 0.0001), demonstrating that Caco-2 TJs proteins could not be hydrolyzed directly by rTsTryp.

### Binding of rTsTryp to Caco-2 cell PAR2 identified by IFA and Co-IP

**Fig 7:**
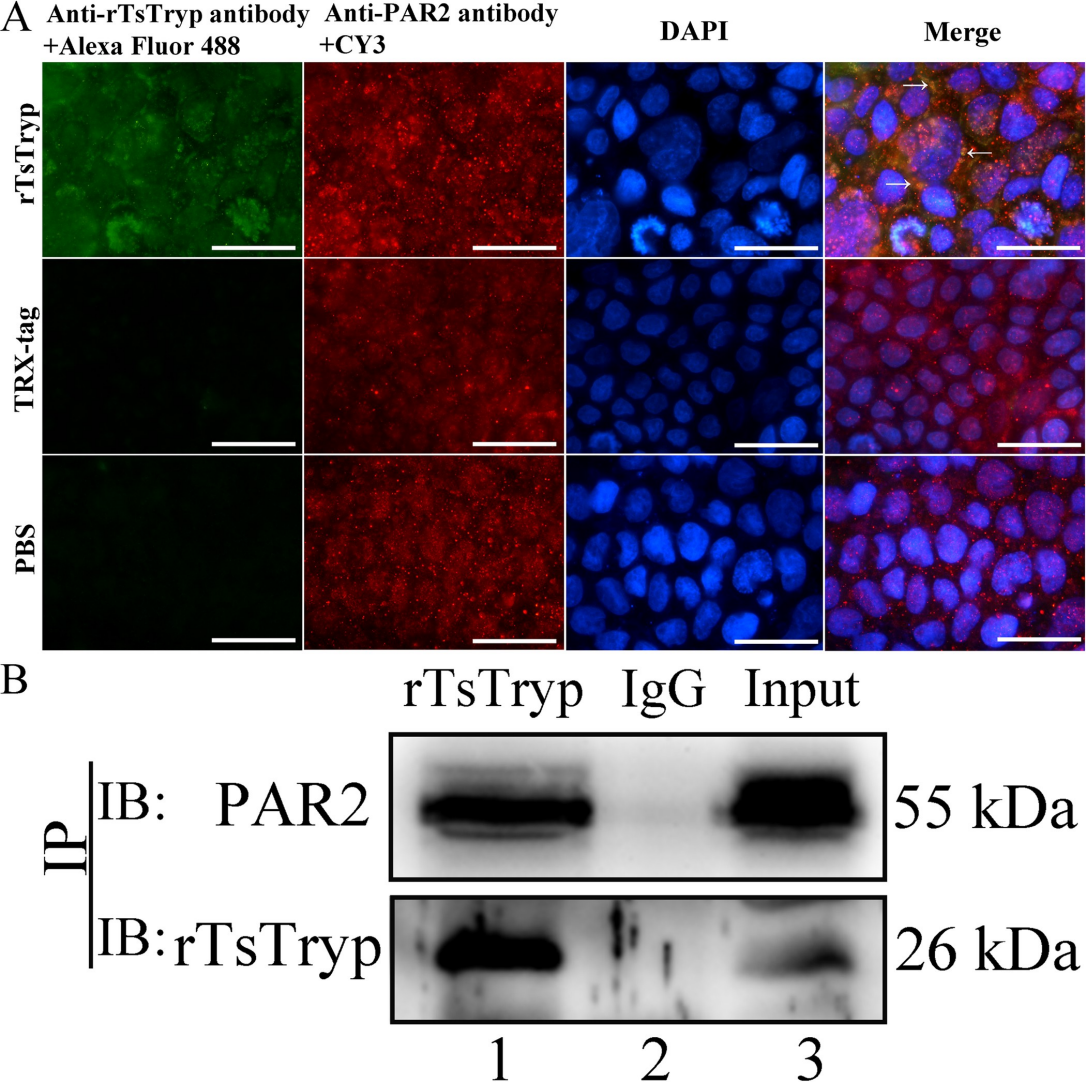
Binding of rTsTryp to PAR2 receptors on Caco-2 monolayer by IFA and Co-IP. **A:** IFA results of rTsTryp binding to PAR2 on Caco-2 monolayer. Caco-2 monolayers were incubated with 20 μg/ml rTsTryp for 2 h, TRX-tag was used as negative control. Anti-rTsTryp antibody and anti-PAR2 antibody were used as the primary antibodies; goat anti-mouse IgG-Alexa Fluor 488 conjugate and CY3-labeled antibodies were used as secondary antibodies. Cell nuclei were stained blue by DAPI. The binding of rTsTryp with PAR2 was localized primarily to the cell membrane (shown by arrows). **B:** Binding of rTsTryp with PAR2 verified by Co-IP. Caco-2 cells were pre-incubated with rTsTryp for 2 h. Anti-rTsTryp antibodies and protein A/G were added, mixed and incubated for 6 h. The bound proteins were separated on SDS-PAGE and transferred to the PVDF membrane. The membrane was probed by anti-rTsTryp antibody and anti-PAR2 antibody. Lane 1: Immune co-precipitation complex (rTsTryp, anti-rTsTryp antibody and PAR2); Lane 2: Normal mouse IgG; Lane 3: Caco-2 cell proteins after incubation with rTsTryp. IP: immunoprecipitation; IB: immunoblotting. Scale bars: 5 μm.

The IFA results showed that when the Caco-2 cell monolayer was incubated with rTsTryp for 2 h, green immunostaining was observed around the cells (e.g., at cell-cell junction) by using anti-rTsTryp antibody and goat anti-mouse IgG-Alexa Fluor 488 conjugate, while natural PAR2 in Caco-2 cells was recognized by an anti-PAR2 antibody and stained red by CY3-labeled antibodies (Fig 7A). The results indicated that both rTsTryp and PAR2 were co-localized at cell–cell junctions, and there was a possible binding of rTsTryp and PAR2. Co-IP revealed that rTsTryp binds to PAR2 in Caco-2 cells. Co-precipitates of rTsTryp and PAR2 were identified using anti-rTsTryp and anti-PAR2 antibodies, respectively; however, these two proteins were not recognized by normal murine IgG (Fig 7B). The results suggested that rTsTryp combined with the PAR2 receptor in Caco-2 cells.

### rTsTryp activating PAR2 down-regulated TJ protein in Caco-2 monolayer

**Fig 8:**
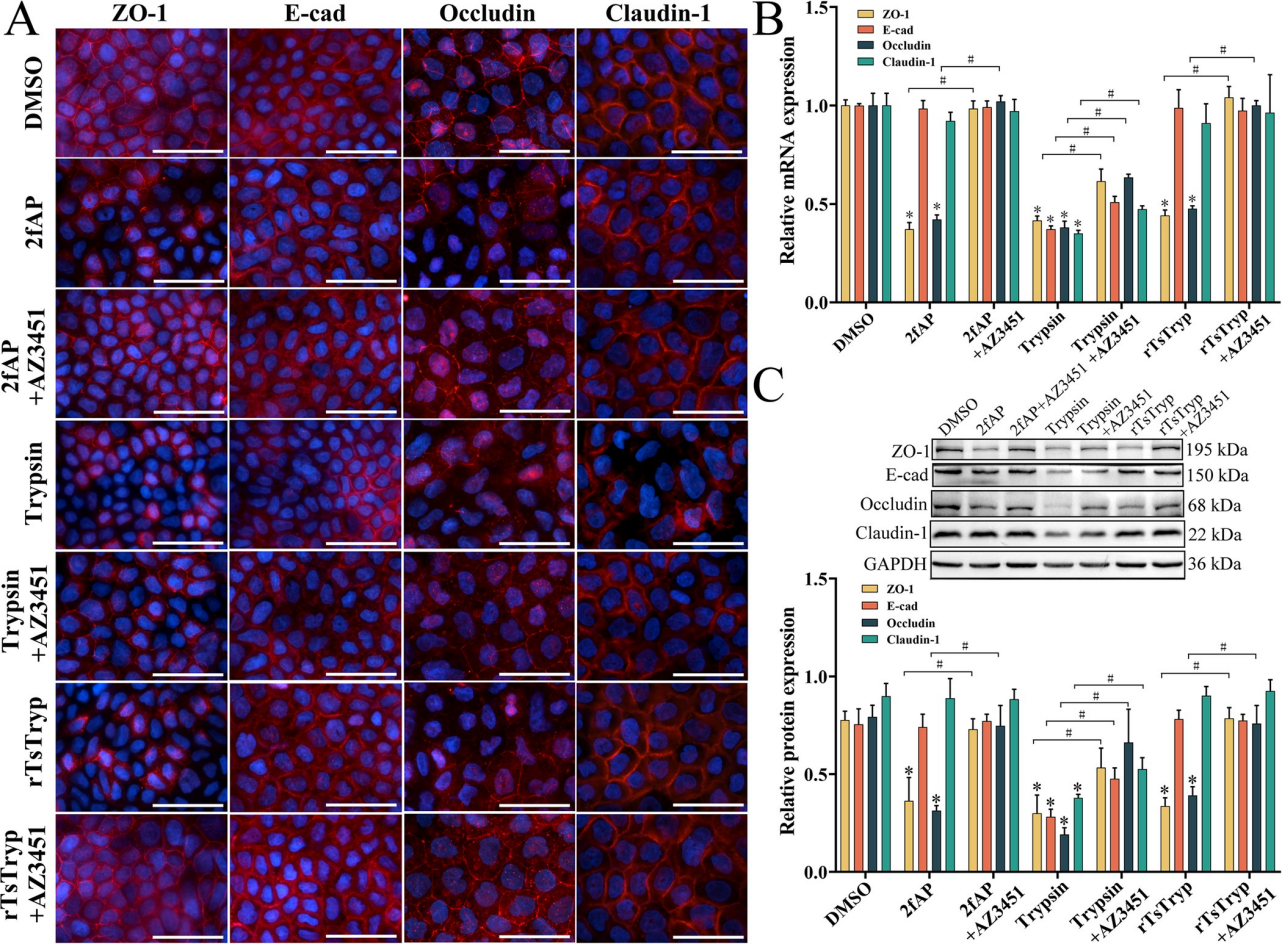
rTsTryp binding to PAR2 reduced expression of ZO-1 and occludin in Caco-2 monolayers. Caco-2 cell monolayer was pretreated with AZ3451 (PAR2 antagonist) for 12 h, then incubated with rTsTryp and PAR2 agonist (2fAP) for 2 h. PAR2 antagonist evidently suppressed and abolished the rTsTryp and PAR2 agonist down-regulating role on ZO-1 and occludin expression. **A:** IFA analysis of ZO-1 and occludin expression in Caco-2 monolayers. **B:** qPCR analysis of ZO-1 and occludin mRNA expression in Caco-2 monolayers. **C:** Western blot analysis of ZO-1 and occludin expression in Caco-2 monolayers. The expression levels of ZO-1, E-cad, occludin and claudin-1 relative to GAPDH were analyzed by gray-level analysis. Each group of data is repeated in triplicate. *P < 0.01 compared to the DMSO group, ^#^*P* < 0.01 between two groups. Scale bars: 5 μm.

The IFA results showed that when Caco-2 monolayers were incubated with rTsTryp and 2fAP, expression of ZO-1 and occludin was obviously reduced or disappeared around the cells; when the monolayer was pretreated with PAR2 antagonist AZ3451 and then incubated with rTsTryp and 2fAP, expression of ZO-1 and occludin was evidently regained and increased compared to the rTsTryp-incubated Caco-2 cells (Fig 8A). Additionally, trypsin significantly reduced the expression of ZO-1, E-cad, occludin, and claudin-1 in Caco-2 cells, whereas the PAR2 antagonist only partially inhibited the reduction of the four TJs proteins, suggesting that trypsin-induced lower expression of the TJs proteins might be in part due to the trypsin hydrolysis of TJs proteins, which could not be inhibited by the PAR2 antagonist.

qPCR results showed that both rTsTryp and the PAR2 agonist (2fAP) had a similar reducing role on the mRNA expression of ZO-1 and occludin in Caco-2 monolayers, and the reduction of rTsTryp and 2fAP on ZO-1 and occludin expression could be abrogated by the PAR2 antagonist (AZ3451) (Fig 8B). In 2fAP-treated Caco-2 cell group, the expression levels of ZO-1 and occludin mRNA was decreased by 62.83 and 57.87% compared to the solvent DMSO group (*F*_ZO-1_ = 613.775, *F*_occludin_ = 236.870, *P* < 0.0001). After Caco-2 cells were treated with rTsTryp, expression levels of ZO-1 and occludin mRNA was decreased by 55.87 and 52.42%, respectively, compared to the DMSO group (*F*_ZO-1_ = 601.326, *F*_occludin_ = 209.098, *P* < 0.0001). However, after pretreatment with the PAR2 antagonist (AZ3451), the expression levels of ZO-1 and occludin mRNA in the 2fAP and rTsTryp groups were restored to the level of the DMSO control group, namely, they were not statistically different from the DMSO control group (2fAP: *F*_ZO-1_ = 0.332, *P* = 0.595; *F*_occludin_ = 0.271, *P* = 0.630. rTsTryp: *F*_ZO-1_ = 1.345, *P* = 0.311; *F*_occludin_ = 0.000, *P* = 0.999).

Western blotting results revealed that rTsTryp and PAR2 agonist (2fAP) also distinctly decreased the expression of ZO-1 and occludin. Compared to the DMSO group, expression of ZO-1 and occludin in 2fAP group was decreased by 53.11 and 60.32% compared to the DMSO respectively (*F*_ZO-1_ = 31.340, *P* < 0.01; *F*_Occludin_ = 168.887, *P* < 0.0001). The ZO-1 and occludin expression levels in rTsTryp group were reduced by 56.48 and 49.05%, respectively (*F*_ZO-1_ = 150.147, *P* < 0.0001; *F*_Occludin_ = 90.076, *P* < 0.01). Moreover, 2fAP- and rTsTryp-reduced-expression of ZO-1, and occludin was also suppressed and abrogated by AZ3451 pretreatment, compared to the 2fAP or rTsTryp alone group (2fAP: *F*_ZO-1_ = 23.448, *F*_Occludin_ = 48.756, *P* < 0.01; rTsTryp: *F*_ZO-1_ = 125.590, *P* < 0.0001; *F*_Occludin_ = 38.402, *P*_Occludin_ < 0.01) (Fig 8C).

### rTsTryp increased paracellular permeability of Caco-2 monolayer

**Fig 9:**
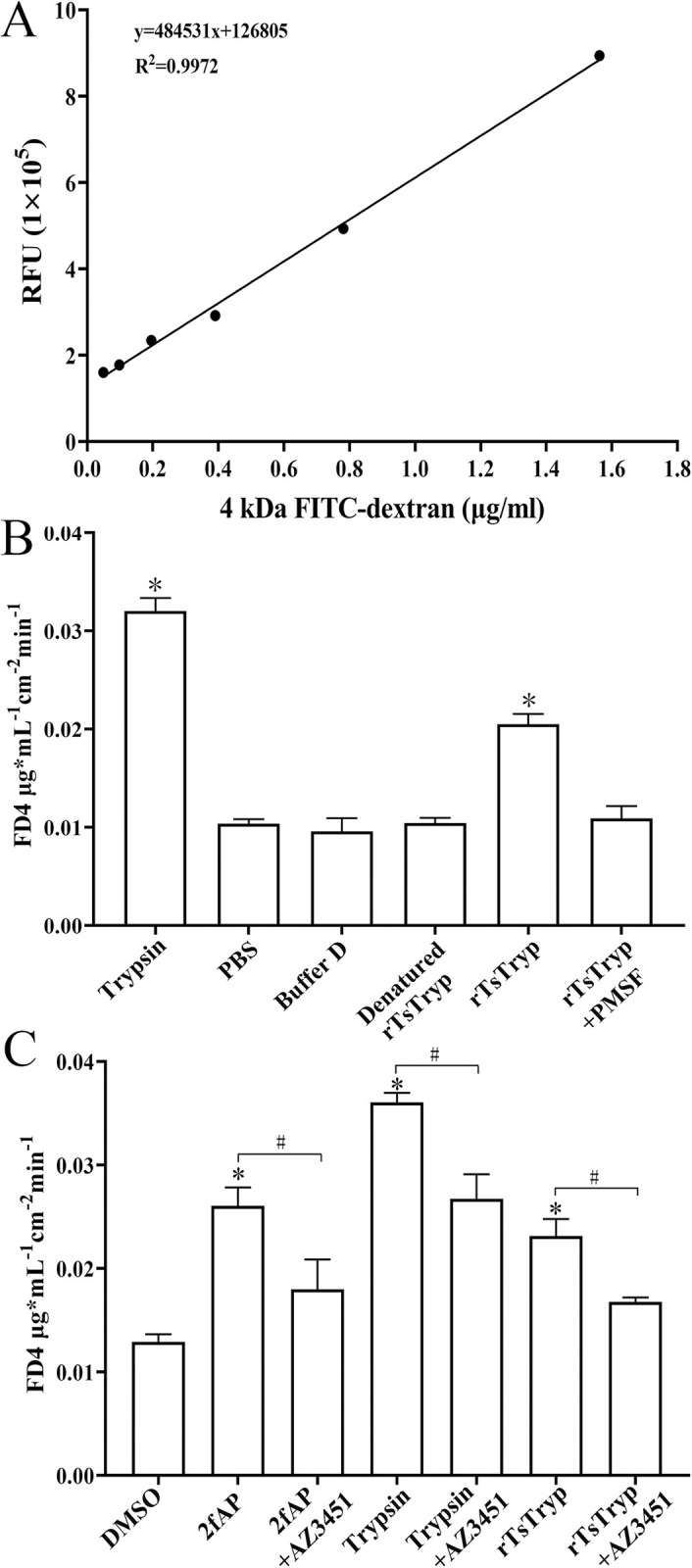
rTsTryp increased paracellular permeability of Caco-2 monolayer. **A:** standard curve of 4 kDa FITC-dextran (0.025–1.6 μg/ml). **B:** The permeability of 4 kDa-FITC dextran was increased after Caco-2 monolayer treated with rTsTryp. **C:** The rTsTryp increasing permeability was evidently suppressed and abrogated by PAR2 antagonist AZ3451. *P < 0.05 compared to PBS/DMSO groups. ^#^*P* < 0.05 between two groups.

The effect of rTsTryp on Caco-2 monolayer integrity was measured by observing changes in the fluorescence intensity of permeated 4 kDa FITC-dextran. First, a standard curve was drawn based on the fluorescence of 0.025–1.6 μg/ml FD4 (Fig 9A). The effects of rTsTryp (trypsin, denatured rTsTryp, or rTsTryp+PMSF) on the permeability of Caco-2 monolayers are shown in Fig 9B. The FD4 influx in the trypsin and rTsTryp groups was 3.2 and 2 folds of the PBS group (*F*_trypsin_ = 472.887, *F*_rTsTryp_ = 115.132, *P* < 0.01), respectively, indicating that rTsTryp significantly increased paracellular permeability and disrupted the integrity of the Caco-2 monolayer barrier. However, the PAR2 antagonist (AZ3451) inhibited and abrogated the rTsTryp’s role, decreased the paracellular permeability. When the cell monolayer was pretreated with AZ3451 and then incubated with rTsTryp, the permeability of dextran was obviously decreased compared to the only rTsTryp group (*F* = 28.106, *P* < 0.05) (Fig 9C). The results demonstrated that the PAR2 antagonist (AZ3451) abrogated rTsTryp damage to Caco-2 monolayer integrity, further suggesting that rTsTryp reduced TJ expression and damaged the monolayer barrier by binding to PAR2 receptor in Caco-2 cells.

### Binding of rTsTryp to PAR2 activated the ERK1/2 pathway

**Fig 10:**
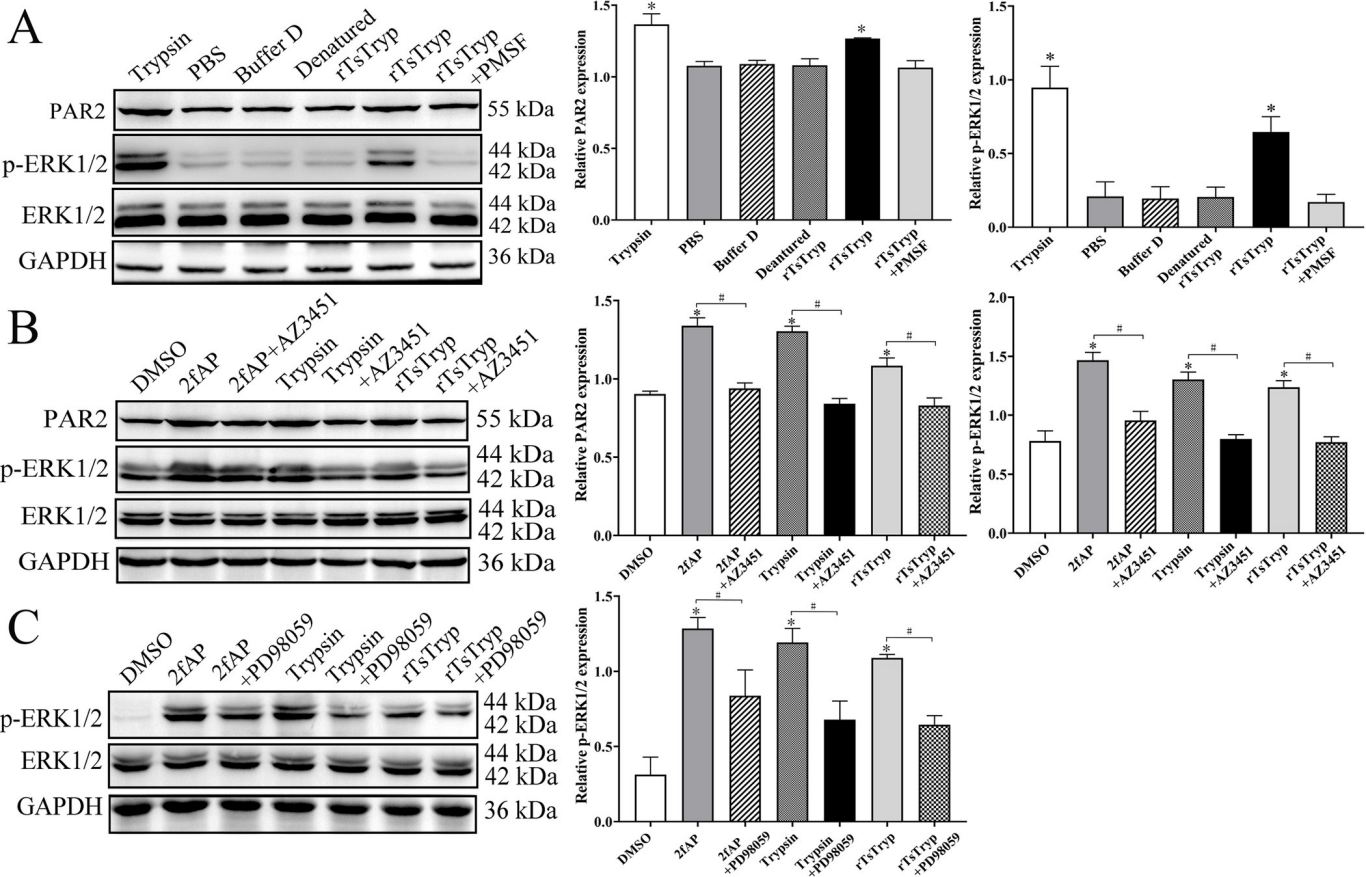
Western blotting of expression levels of PAR2 and p-ERK1/2 in Caco-2 cells after rTsTryp treatment. **A:** rTsTryp increased expression level of PAR2 and p-ERK1/2 in Caco-2 cells, but denatured rTsTryp and rTsTryp+PMSF did not. **B:** PAR2 antagonist AZ3451 inhibited and abolished the rTsTryp activating PAR2 and p-ERK1/2 expression. **C:** ERK1/2 pathway inhibitor PD98059 suppressed and abrogated rTsTryp activating p-ERK1/2 expression level. GAPDH was used as an internal control. Densitometry of the protein bands was analyzed by gray-level analysis. *P < 0.01 compared to the PBS/DMSO groups, ^#^*P* < 0.01 between two groups.

After Caco-2 monolayer was incubated with rTsTryp for 2 h, the PAR2 expression level was increased by 21.12% compared to the PBS group (*F* = 84.647, *P* < 0.01). The level of phosphorylated ERK1/2 (p-ERK1/2) was increased by 209.59% compared to the PBS group (*F* = 27.760, *P* < 0.01) (Fig 10A). However, inactivated rTsTryp and PMSF-treated rTsTryp did not increase the expression levels of PAR2 and p-ERK1/2 (*P* ˃ 0.05). When Caco-2 monolayers were pretreated with PAR2 antagonist (AZ3451) and then incubated with rTsTryp, compared to the only rTsTryp group, the expression levels of PAR2 and p-ERK1/2 was decreased by 23.42% (*F* = 40.420, *P* < 0.01) and 37.59% (*F* = 125.179, *P* < 0.0001), respectively (Fig 10B), indicating that PAR2 antagonist inhibited and abolished the rTsTryp activating PAR2 and p-ERK1/2 expression. Additionally, when Caco-2 monolayer was pretreated with ERK1/2 pathway inhibitor PD98059 and then incubated with rTsTryp, the expression level of p-ERK1/2 was reduced by 40.92% (*F* = 140.127, *P* ˂ 0.0001) relative to the rTsTryp alone group (Fig 10C), suggesting that PD98059 suppressed the activation of ERK1/2 pathway and abrogated the rTsTryp activating ERK1/2 pathway. The results further confirmed that rTsTryp bound to the PAR2 receptor in Caco-2 cells and activated the ERK1/2 pathway.

### rTsTryp mediated larval invasion of Caco-2 monolayer via binding to PAR2 and activating ERK1/2 pathway

**Fig 11:**
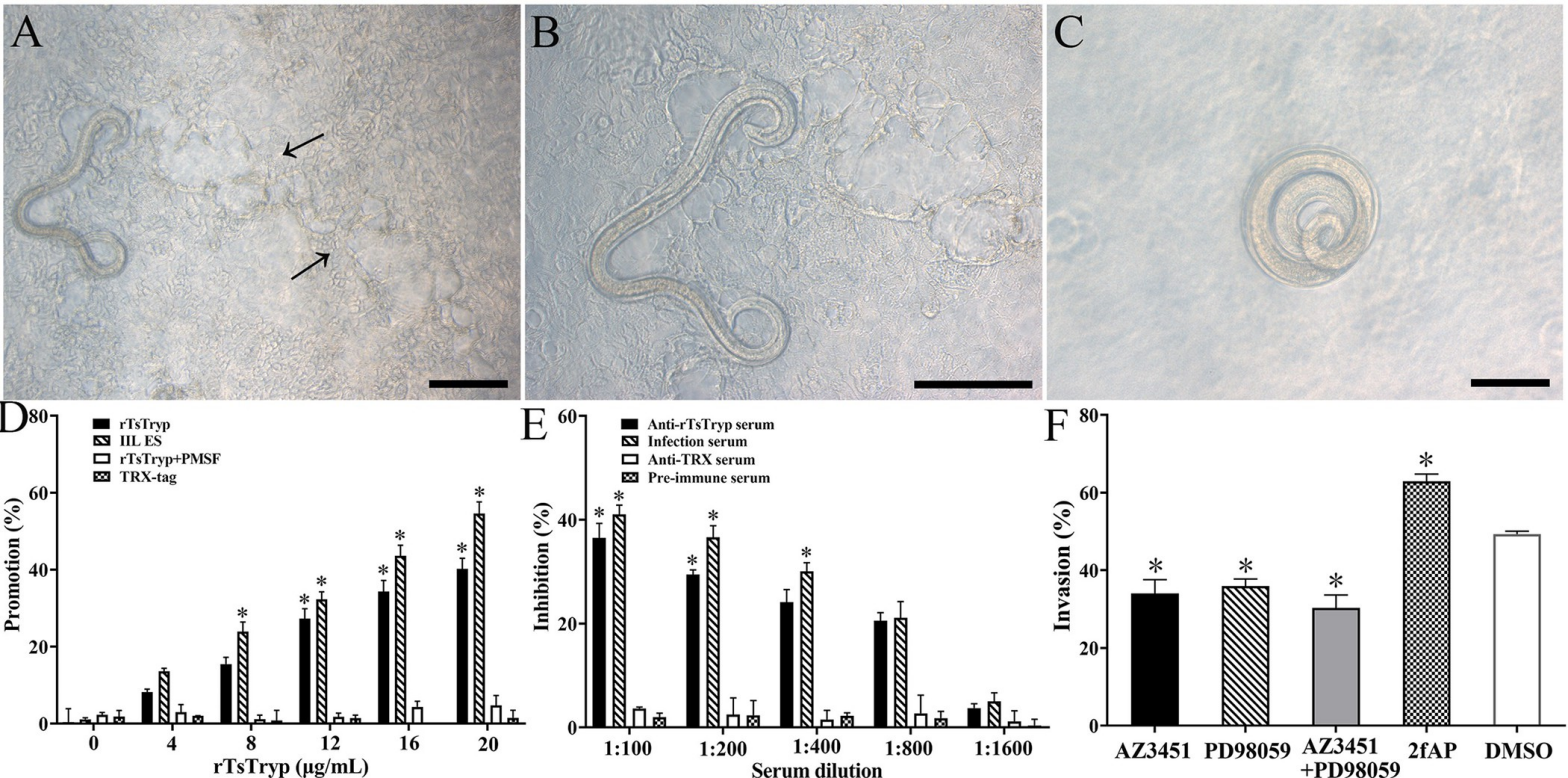
rTsTryp mediated larval invasion of Caco-2 monolayer via binding to PAR2 and activating ERK1/2 pathway. **A** and **B:** *T*. *spiralis* IIL invaded Caco-2 cell monolayer, arrows indicate larval migratory traces; **C:** non-invaded larva was spirally coiled on the surface of Caco-2 monolayer; **D:** Promotion of rTsTryp on larval invasion of Caco-2 monolayer; **E:** Inhibition of anti-rTsTryp antibody on larval invasion of Caco-2 monolayer. **F:** PAR2 agonist 2fAP promoted larval invasion; PAR2 antagonist (AZ3451) and ERK1/2 inhibitor (PD98059) suppressed the larval invasion. *P < 0.05 compared to the TRX tag, pre-immune serum or DMSO group. Scale-bars: 100 μm.

The *in vitro* larval invasion test showed that IIL penetrated the Caco-2 monolayer and showed obvious migratory traces (black arrow) (Fig 11A and 11B). The non-penetrated IIL was suspended in the medium or curled spirally on the monolayer surface (Fig 11C). When the rTsTryp was added to the medium, rTsTryp had an obvious promotion role on the larval invasion; when 12, 16 and 20 μg/ml rTsTryp was used, the promotion rate of IIL invasion was 27.32, 34.37 and 40.24%, respectively relative to the PBS group (χ^2^_12_ = 4.393, χ^2^_16_ = 5.725, *P* < 0.05; χ^2^_20_ = 8.164, *P* < 0.01), and rTsTryp promotion was positively correlated with the concentrations of rTsTryp (*F* = 5.165, *r* = 0.788, *P* < 0.05), but PMSF-treated rTsTryp and TRX tag protein did not have any promotion on larval invasion (Fig 11D), suggesting that rTsTryp mediating larval invasion was likely related to the enzymatic activity of rTsTryp. Anti-rTsTryp antibodies blocked IIL invasion in a dose-dependent manner (*F* = 11.854, *r* = 0.913, *P* < 0.01). When 1:200 dilutions of anti-rTsTryp serum were used, the invasion was inhibited by 29.47% compared to the PBS group (*χ*^2^ = 4.723, *P* < 0.05) (Fig 11E). Moreover, the PAR2 agonists (2fAP) facilitated larval invasion with an increased invasion of 27.54% compared to the solvent DMSO group (χ^2^ = 3.952, *P* < 0.05); PAR2 antagonist (AZ3451), ERK1/2 inhibitors (PD98059), and AZ3451+PD98059 inhibited larval invasion; the invasion rate was decreased by 30.98, 27.16 and 38.62%, respectively (*χ*^2^_AZ3451_ = 4.966, *χ*^2^_PD98059_ = 4.044, *P* < 0.05; *χ*^2^_AZ3451+PD98059_ = 8.194, *P* < 0.01), compared to the DMSO group (Fig 11F).

### AZ3451 and PD98059 reduced enteral IIL and adult burdens in mice after challenge

**Fig 12:**
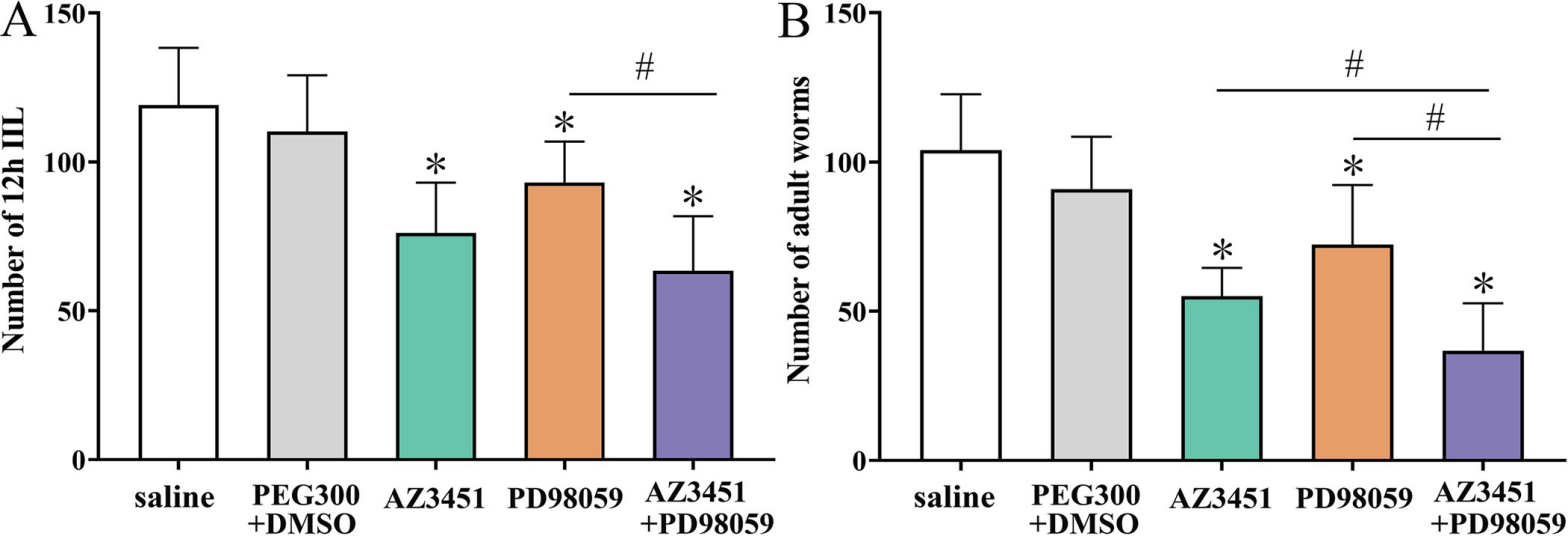
AZ3451 and PD98059 reduced the intestinal IIL and adult worm burden at 12 h and 5 d post infection. **A:** 12 h IIL burden (n = 10); **B:** Intestinal adult worm burden (n = 10). The data of various groups are shown as the mean ± SD. *P < 0.01 relative to the saline group, ^#^*P* < 0.01 relative to the AZ3451+PD98059 group.

All the infected mice were sacrificed at 12 hpi and 5 dpi, respectively. Enteral IIL worms and adult worms were collected and numbered. The results showed that the IIL burdens of AZ3451, PD98059, and AZ3451+PD98059 groups were reduced by 36.02 (*F* = 28.034, *P* < 0.0001), 21.83 (*F* = 12.072, *P* < 0.01) and 46.77% (*F* = 43.839, *P* < 0.0001), respectively, compared to the saline group (Fig 12A). The IIL burden of AZ3451+PD98059 group was reduced by 31.90% compared to the PD98059 group alone (*F* = 16.794, *P* < 0.0001), but the IIL burden was not statistically different between AZ3451+PD98059 and AZ3451 groups (*P* > 0.05). At 5 dpi, compared to the saline group, the adult burdens of AZ3451, PD98059, and AZ3451+PD98059 groups were decreased by 47.01 (*F* = 49.748, *P* < 0.0001), 30.48 (*F* = 13.390, *P* < 0.01) and 64.62% (*F* = 74.936, *P* < 0.0001), respectively (Fig 12B). The adult burden of AZ3451+PD98059 group was significantly lower than the only AZ3451 and PD98059 alone groups, as demonstrated the adult burden of AZ3451+PD98059 group being reduced by 33.23 (*F* = 9.059, *P* < 0.01) and 49.10% (*F* = 19.337, *P* < 0.0001), respectively, compared to the only AZ3451 and PD98059 alone groups. The results suggested that the PAR2 antagonist AZ3451 and the ERK1/2 inhibitor PD98059 significantly suppressed IIL invasion of host gut mucosa and larval development, and the inhibitory effect of the AZ3451+PD98059 group was higher than that of AZ3451 and PD98059 alone.

### AZ3451 and PD98059 reduced PAR2 and p-ERK1/2 levels in infected mice

**Fig 13:**
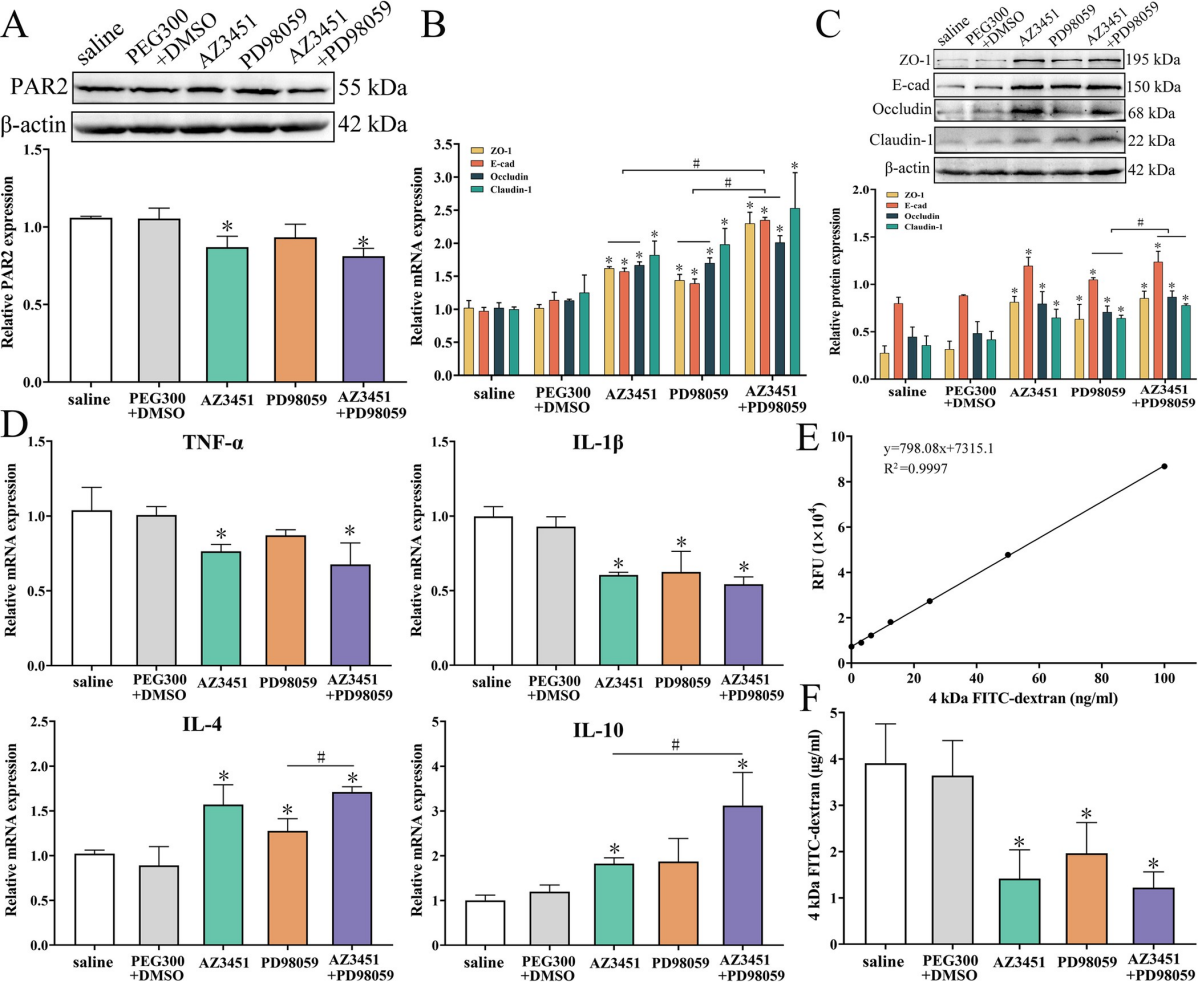
Changes of PAR2, TJs proteins, intestinal inflammatory cytokines and permeability in *T*. *spiralis*-infected mouse intestine. **A:** Western blot analysis of AZ3451 inhibiting PAR2 expression. PAR2 expression in intestinal mucosa of the AZ3451 and AZ3451+PD98059 groups was obviously decreased. **B:** qPCR analysis of AZ3451 and PD98059 increasing mRNA expression of TJs proteins. **C:** Western blot analysis of AZ3451 and PD98059 increasing expression of TJs proteins. AZ3451 and PD98059 increased the expression of TJs proteins in infected murine intestine. The expression levels of PAR2, ZO-1, E-cad, occludin and claudin-1 relative to β-actin were analyzed by gray-level analysis. **D:** AZ3451 and PD98059 alleviated intestinal inflammation caused by *T*. *spiralis* infection. Two inhibitors inhibited transcription of gut pro-inflammatory cytokines (TNF-α and IL-1β) and up-regulated transcription of gut anti-inflammatory cytokines (IL-4 and IL-10) in infected mice. GAPDH was used as an internal reference to analyze the transcription level of cytokines. **E:** Standard curves of 0–100 ng/ml 4 kDa FITC-dextran. **F:** AZ3451 and PD98059 abrogated intestinal permeability increase caused by *T*. *spiralis* infection, as demonstrated by intestinal FD-4 flux evidently reduced. Data of each test are repeated in triplicate. *P < 0.05 compared to the saline group. ^#^*P* < 0.05 relative to the AZ3451+PD98059 group.

Western blotting results showed that the PAR2 expression level in infected murine intestines of the PAR2 antagonist AZ3451 and AZ3451+PD98059 groups was decreased by 17.87 (*F* = 21.489, *P* < 0.05) and 23.53% (*F* = 67.106, *P* < 0.01), respectively, compared to the saline group (Fig 13A). However, the PAR2 expression level in the PD98059 group was not statistically different from that in the saline and solvent control groups (*P* > 0.05), indicating that the PAR2 antagonist AZ3451 significantly inhibited PAR2 expression in the infected murine intestine.

Immunohistochemical staining revealed that the phosphorylation level of ERK1/2 (p-ERK1/2) in the intestinal tissues of the AZ3451, PD98059, and AZ3451+PD98059 groups was significantly lower than that in the saline and PEG300+DMSO groups (S5 Fig). Compared with the saline group, the p-ERK1/2 levels of the AZ3451, PD98059, and AZ3451+PD98059 were decreased by 48.37 (*F* = 15.774, *P* < 0.05), 51.57 (*F* = 18.326, *P* < 0.05), and 73.16% (*F* = 36.465, *P* < 0.01), respectively. Moreover, the p-ERK1/2 levels in AZ3451+PD98059 group were significantly lower than the individual AZ3451 and PD98059 groups, as decreased by 48.01 (*F* = 25.222, *P* < 0.01) and 44.57% (*F* = 22.011, *P* < 0.01), respectively. The results further showed that AZ3451 inhibited the activation and expression of PAR2, and PD98059 suppressed the phosphorylation level of the ERK1/2 in infected murine intestine, further demonstrating that TsTryp binds to PAR2 and activates the ERK1/2 pathway in the gut mucosa at the intestinal stage of *T*. *spiralis* infection.

### AZ3451 and PD98059 up-regulating TJs expression in infected murine intestine

The qPCR results showed that the transcription levels of ZO-1, E-cad, occludin, and claudin-1 in the AZ3451, PD98059, and AZ3451+PD98059 groups were evidently increased relative to the saline group (Fig 13B). In AZ3451 group, the transcription levels of ZO-1, E-cad, occludin, and claudin-1 were increased by 58.55, 61.53, 63.46, and 82.29%, respectively (*F*_ZO-1_ = 85.572, *P* < 0.01; *F*_E-cad_ = 207.329, *P* < 0.0001; *F*_occludin_ = 140.529, *P* < 0.0001; *F*_claudin-1_ = 27.734, *P* < 0.05).The transcription levels of ZO-1, E-cad, occludin, and claudin-1 in the PD98059 group were increased by 40.82, 42.92, 66.86 and 98.35%, respectively (*F*_ZO-1_ = 26.603, *P* < 0.01; *F*_E-cad_ = 72.245, *P* < 0.01; *F*_occludin_ = 114.307, *P* < 0.0001; *F*_claudin-1_ = 30.100, *P* < 0.05). In the AZ3451+PD98059 group, the transcription levels of ZO-1, E-cad, occludin, and claudin-1 were increased by 125.02, 141.25, 97.33, and 153.07%, respectively (*F*_ZO-1_ = 122.429, *F*_E-cad_ = 1248.929, *F*_occludin_ = 179.888, *P* < 0.0001; *F*_claudin-1_ = 14.604, *P* < 0.05). Moreover, the transcription levels of ZO-1, E-cad, and occludin in the AZ3451+PD98059 group were significantly higher than those in individual AZ3451 or PD98059 group (*F*_ZO-1_ = 51.092, *F*_E-cad_ = 281.036, *P* < 0.0001; *F*_Occludin_ = 17.485, *P* < 0.01).

Western blotting results revealed that AZ3451 and PD98059 significantly up-regulated the expression levels of TJs proteins (Fig 13C). Compared to the saline group, the expression of ZO-1, E-cad, occludin, and claudin-1 in AZ3451 group was increased by 195.99, 49.78, 78.16 and 81.50%, respectively (*F*_ZO-1_ = 92.538, *F*_E-cad_ = 39.067, *P* < 0.01; *F*_occludin_ = 13.535, *F*_claudin-1_ = 14.381, *P* < 0.05). The expression of ZO-1, E-cad, occludin, and claudin-1 in PD98059 group was increased by 130.29, 31.41, 58.42 and 79.94% (*F*_ZO-1_ = 12.746, *P* < 0.05; *F*_E-cad_ = 39.977, *P* < 0.01; *F*_occludin_ = 13.897, *P* < 0.05; *F*_claudin-1_ = 22.836, *P* < 0.01), respectively. In the AZ3451+PD98059 group, the expression of ZO-1, E-cad, occludin, and claudin-1 was increased by 210.79, 54.97, 94.12, and 118.15%, respectively (*F*_ZO-1_ = 88.485, *F*_E-cad_ = 34.868, *F*_occludin_ = 36.132, *F*_claudin-1_ = 53.707, *P* < 0.01), respectively. Furthermore, the expression of E-cad, occludin, and claudin-1 in the AZ3451+PD98059 group was significantly higher than that in the PD98059 group (*F*_E-cad_ = 8.300, *F*_occludin_ = 9.248, *P* < 0.05; *F*_claudin-1_ = 45.004, *P* < 0.01).

The results suggested that the PAR2 antagonist AZ3451 inhibited the expression of PAR2, reduced the phosphorylation level of ERK1/2, and up-regulated TJs expression, whereas the ERK1/2 inhibitor PD98059 directly inhibited the phosphorylation of ERK1/2 and increased the expression of TJs. The effect of AZ3451+PD98059 was greater than that of either single AZ3451 or PD98059 alone. These results verified that TsTryp damaged Caco-2 monolayer integrity, further suggesting that rTsTryp binding to PAR2 activated the ERK1/2 pathway, reduced TJs expression, damaged gut epithelium integrity, and thereby mediated IIL invasion of the gut mucosa.

### AZ3451 and PD98059 alleviated intestinal inflammation of infected mice

The transcriptional levels of inflammatory cytokines in the intestines of infected mice were assessed by qPCR. qPCR results showed that the transcription levels of pro-inflammatory cytokines TNF-α and IL-1β in the AZ3451 and AZ3451+PD98059 groups were significantly lower than those in the saline group (TNF-α: *F*_AZ3451_ = 8.955, *P* < 0.05; *F*_AZ3451 +PD98059_ = 8.997, *P* < 0.05; IL-1β: *F*_AZ3451_ = 102.217, *P* < 0.01; *F*_AZ3451 +PD98059_ = 93.181, *P* < 0.01), and only the transcription levels of IL-1β in the PD98059 group were lower than those in the saline group (*F*_PD98059_ = 17.889, *P* < 0.05) (Fig 13D). Moreover, the levels of anti-inflammatory cytokines IL-4 and IL-10 in the AZ3451 and AZ3451+PD98059 groups were significantly increased relative to those in the saline group (IL-4: *F*_AZ3451_ = 17.788, *P* < 0.05, *F*_AZ3451+PD98059_ = 282.205, *P* < 0.0001; IL-10: *F*_AZ3451_ = 52.019, *P* < 0.01; *F*_AZ3451 +PD98059_ = 14.562, *P* < 0.05), and the transcription level of IL-4 in the PD98059 group was higher than that in the saline group (*F*_PD98059_ = 9.495, *P* < 0.05). Additionally, the IL-4 transcription level in the AZ3451+PD98059 group was significantly higher than that in the PD98059 group (*F* = 25.373, *P* < 0.01), and the IL-10 level in the AZ3451+PD98059 group was higher than that in the AZ3451 alone group (*F* = 8.949, *P* < 0.05). These results further indicated that AZ3451 and PD98059 inhibited the *T*. *spiralis* invasion of the gut epithelium, reduced the expression of pro-inflammatory cytokines, and up-regulated the expression of anti-inflammatory cytokines, resulting in amelioration of intestinal inflammation caused by *T*. *spiralis* infection.

### AZ3451 and PD98059 reduced intestinal permeability in infected mice

Changes in intestinal TJ proteins directly affect intestinal permeability, and 4 kDa FITC-dextran (FD-4) is commonly used to measure permeability *in vivo*. To test whether AZ3451 and PD98059 decrease the increased intestinal permeability caused by *T*. *spiralis* infection, mice were pretreated with AZ3451, PD98059 and AZ3451+PD98059, followed by *T*. *spiralis* challenge. The results showed that at 4 h after FD-4 administration, the FD-4 fluxes in AZ3451, PD98059 and AZ3451+PD98059 groups were significantly lower than the saline group (Fig 13E and 13F), which were only 0.36 (*F* = 27.938, *P* < 0.01), 0.50 (*F* = 16.244, *P* < 0.01) and 0.31 folds (*F* = 42.988, *P* < 0.0001) of the saline group, respectively. The FD-4 fluxes of AZ3451+PD98059 were not significantly different from those of the individual AZ3451 and PD98059 alone groups (*P* ˃ 0.05). These results suggest that AZ3451 and PD98059 abrogated the increase of intestinal permeability resulting from *T*. *spiralis* infection, and also verified that AZ3451 and PD98059 inhibited the expression of PAR2 and p-ERK1/2, thereby up-regulating and restoring the expression of gut epithelial TJs proteins and regaining gut epithelial integrity and barrier function.

## Discussion

The gastrointestinal tract provides an opportunity for contact between the body and the external environment; it selectively absorbs the nutrients and water needed by the body and blocks the penetration of harmful substances such as parasites, bacteria, and toxins. This selective permeable mode is achieved through tight junction complexes, which regulate paracellular permeability [64]. The IECs and apical junction complex (AJC) constitute the natural physical barrier of intestinal epithelium, and the AJC is mainly composed of TJs, adhesion junctions (AJs), and desmosomes. TJs proteins include occludin, claudin-1, JAMs, and polarity proteins, such as ZO-1. AJs proteins include E-cad and nectins [65]. The TJs between IECs prevent the invasion of luminal macromolecules and pathogens, and protect against inflammation and infection. Damage to TJs and AJC proteins inevitably leads to the breakdown of the intestinal barrier. The destruction of intestinal barrier structure results in direct invasion of pathogens and harmful substances, and leads to the occurrence of intestinal diseases [66].

Interactions between intestinal pathogens and IECs disrupt the intestinal barrier, results in abnormal intestinal function and mucosal inflammation [64]. Pathogens destroy TJs proteins by their secreting proteases or binding directly to IECs. In intestinal parasite infection, proteases secreted by parasites play a vital role in destroying intestinal barrier function more than the mechanical damage [67]. Recent studies showed that *Fusobacterium nucleatum* (an oral and intestinal pathogenic bacterium) damaged intestinal epithelial integrity and increased intestinal epithelial permeability through regulating the distribution and expression of TJs proteins ZO-1 and occludin, and increasing the secretion of inflammatory cytokines (TNF-α, INF-γ, and IL-1β) which increased intestinal inflammation and exacerbated colitis [68]. In intestinal parasitic protozoan infection, the paracellular permeability of Caco-2 monolayer was increased after adhesion of *Giardia duodenalis* trophozoites, and confocal microscopy showed that the disturbance of TJs, AJs and desmosome junctions resulted in the increase of paracellular permeability [69]. In *T*. *spiralis* infection, the serine protease in the ML ES proteins obviously increased the permeability of the cell monolayer through reducing the TJs expression [40]. Our previous studies indicated that serine proteases and cysteine proteases in IIL ES proteins disrupted intestinal epithelial integrity by down-regulating expression of E-cad, occludin and claudin-1 and up-regulating claudin-2 expression [18]. The results suggested that serine proteases in IIL ES proteins are the key invasive factors for *T*. *spiralis* invasion of gut mucosa.

In this study, TsTryp, a serine protease with a trypsin-like domain, was cloned and expressed. TsTryp is a secretory protease expressed at various *T*. *spiralis* developmental stages with the highest expression in the IIL invasive stage. The results of IFA, ELISA, and western blotting showed that rTsTryp bound to the IECs, and the binding sites were mainly localized in cytomembrane and a litter in cytoplasm of the IEC. The presence of rTsTryp in IEC cytoplasm might be due to the endocytosis of rTsTryp protein by living IEC cells. Endocytosis is a cellular mechanism by which cells internalize substances in the external environment, including proteins, liquids, electrolytes, microorganisms, and some large molecules, which undergo certain breakdown into smaller elements for cell use or elimination [70]. Previous studies showed that exogenous protein aggregation on cell surface triggered endocytosis to maintain plasma membrane proteostasis [71]. Therefore, the localization of a little rTsTryp in IEC cytoplasm in this study might be due to the aggregation of rTsTryp protein around IECs, which leads to the endocytosis of some rTsTryp protein to maintain the cell plasma membrane proteostasis. Purified rTsTryp has the enzymatic activity of natural trypsin on the substrate BAEE and gelatin. However, rTsTryp could not directly degrade TJs proteins (ZO-1, E-cad, occludin, and claudin-1) *in vitro*, indicating that rTsTryp did not have the capacity to directly hydrolyze TJs proteins. Even so, when Caco-2 monolayers were incubated with rTsTryp for 2 h, obvious fractures in the reticular structural junctions of ZO-1 and occludin at cell-cell junctions were observed. The results of qPCR and western blotting showed that the expression levels of ZO-1 and occludin in Caco-2 monolayers were significantly reduced. rTsTryp down-regulation of ZO-1 and occludin could be inhibited by the serine protease inhibitor PMSF. The results suggested that rTsTryp significantly down-regulated the expression of TJs proteins (ZO-1 and occludin), impairing the integrity of the intestinal epithelial barrier. The down-regulation of rTsTryp might be related to its enzymatic activity [23, 40].

rTsTryp reduced the expression of TJs (ZO-1 and occludin), but it could not directly hydrolyze TJs proteins, suggesting that rTsTryp damaging the integrity of gut epithelial barrier was not resulted from the rTsTryp direct hydrolysis on TJs proteins, it was likely due to rTsTryp down-regulating the expression of TJs proteins in Caco-2 monolayers through certain mechanisms. The proteases secreted by intestinal pathogens not only directly hydrolyze the extracellular matrix, but also act as a signaling molecule via specific receptor [64]. Trypsin-like serine proteases impairing the integrity of intestinal epithelium barrier is likely because it activated the PAR2 receptor to cause the opening of functional TJs proteins [72, 73]. PAR2, belonging to the G-protein-coupled receptors family, is a seven-transmembrane protein receptor, and it is a receptor activated by trypsin, is expressed throughout the gastrointestinal tract, particularly highly expressed in the IECs [25]. Trypsin and tryptase cleave PAR2 at the extracellular N-terminus to expose tethered ligand domains, thereby activating the PAR2 receptor and the downstream pathway. Synthetic peptides based on the activation sequence of tethered ligand domains, such as the PAR2 agonist (2fAP), are also able to activate PAR2 by binding directly to its receptor [27, 74]. Activation of PAR2 causes intestinal inflammation, including vascular dilatation, cytokine up-regulation, recruitment of immune cells, and changes in the intestinal barrier [75]. Previous studies showed that the *Vibrio cholerae*-derived AT1002 was able to competitively bind to 50% of the H-labeled PAR2 ligand, indicating that the AT1002 effect on intestinal permeability was PAR2-dependent [76]. Although the direct cleavage of PAR2 receptor by TsTryp was not confirmed in this study, our results indicated that rTsTryp bound with PAR2 by IFA co-localization and Co-IP experiments and activated the PAR2 receptor, as demonstrated by the increased PAR2 expression level after Caco-2 cells were incubated with rTsTryp. The results suggested that rTsTryp with trypsin activity possibly cleaved the N-terminal sequence of PAR2 receptor to activate PAR2 [56, 74]. Activated PAR2 modulates a variety of cellular functions. The PAR2 agonist (2fAP) and trypsin can induce redistribution of TJs proteins on the surface of colonocytes to increase gut epithelial permeability.

Our results showed that when Caco-2 monolayers were incubated with 2fAP and rTsTryp, PAR2 was activated and the cytoskeleton was regulated, as demonstrating that ZO-1 and occludin of TJs proteins were evidently decreased at the gene and protein expression levels, and the contents of ZO-1 and occludin at cell-cell junctions were also clearly reduced. However, rTsTryp had no obvious effect on E-cad and claudin-1 expression. When Caco-2 monolayers were treated with trypsin, the contents of all TJs proteins (ZO-1, E-cad, occludin, and claudin-1) were evidently decreased, which is likely due to the combination of trypsin’s direct hydrolysis and PAR2 activation to down-regulate TJs expression. The results of this study also showed that the PAR2 antagonist evidently suppressed and abolished the rTsTryp- and 2fAP-down-regulating roles on ZO-1 and occludin expression, suggesting that rTsTryp binding to PAR2 reduced the expression of TJs proteins in Caco-2 monolayers. Previous studies showed that when PAR2 agonists were used on the basolateral surface of colonocytes, they decreased transepithelial resistance, increased transepithelial flux of macromolecules, and induced reallocation of ZO-1 and occludin, but they had no obvious effect on E-cad and claudin-1 [77]. Our results demonstrated that rTsTryp could bind especially to the PAR2 receptor, activated and up-regulated the expression of PAR2 in Caco-2 monolayers. In *T*. *spiralis*-infected mice, PAR2 expression was increased, and the expression of TJs proteins (ZO-1 and occludin) was significantly decreased in infected mice [25].

In the present study, we investigated the effect of rTsTryp on the paracellular permeability of Caco-2 monolayers. When added to the surface of the Caco-2 monolayer, rTsTryp distinctly increased the paracellular permeability. However, when Caco-2 monolayers were pretreated with the PAR2 antagonist AZ3451, paracellular permeability was decreased, suggesting that AZ3451 suppressed and abolished the rTsTryp down-regulation on TJs proteins. The results indicated that rTsTryp activated PAR2 receptors to down-regulate the expression of intracellular TJs proteins, thereby increased paracellular permeability. The results are similar to the previous *in vivo* experiment; trypsin or injection of PAR2 agonists in mice resulted in gut luminal PAR2 activation, and then increased intestinal epithelial permeability through cytoskeletal contraction [78]. In contrast to wild type mice, perfusion of trypsin in colon lumen of PAR2-deficient mice did not increase intestinal permeability [79]. Nevertheless, the mechanism of interaction between activated PAR2 and cytoskeletal contraction remains to be elucidated.

Analysis of the MAPK signaling pathway revealed that PAR2 mediated changes in paracellular permeability. Stimulation of PAR2 leads to the phosphorylation of ERK1/2, which has been shown to play a crucial role in gut inflammatory responses [80, 81]. Physiological regulation of gut epithelial TJs is related to ERK1/2-dependent myosin light-chain phosphorylation. In an *in vitro* model of the IECs, apical and basolateral PAR2 could be activated by the PAR2 agonists and promoted the activation of ERK1/2, whereas the phosphorylation of ERK1/2 induced by basolateral PAR2 activation promoted activation of F-actin and thus led to the reorganization of TJs proteins [56]. To verify that the ERK1/2 signaling pathway is involved in the expression change of TJs proteins (ZO-1 and occludin) caused by rTsTryp-induced PAR2 activation, the expression levels of PAR2 and TJs proteins in Caco-2 monolayers incubated with rTsTryp were ascertained in this study. The results showed that rTsTryp, trypsin, and the PAR2 agonist (2fAP) activated PAR2 and caused the phosphorylation of ERK1/2, whereas the PAR2 antagonists (AZ3451) and the ERK1/2 inhibitor PD98059 inhibited the phosphorylation of ERK1/2, indicating that the activation of ERK1/2 is necessary for PAR2-induced increase in permeability. Other studies also revealed that PAR2 in colonocytes activated the downstream ERK1/2 pathway, and its phosphorylation could be abrogated by the PAR2 antagonist AZ3451 [82, 83]. ERK1/2 activation is involved in the expression of ZO-1 and occludin in the small intestine [84]. ERK1/2 could also regulate epithelial permeability, possibly through affecting the cytoskeleton or promoting TJs protein phosphorylation. However, for cytoskeleton and TJs protein regulation, activated ERK1/2 must remain in the cytosol rather than being displaced into the nucleus [56, 77].

Activation of PAR2 is involved in various intestinal inflammatory diseases [24]. The PAR2 antagonist AZ3451 was shown to bind to a remote allosteric site outside the helical bundle of the PAR2 receptor, and AZ3451 binding prevented the structural rearrangement required for PAR2 receptor activation and downstream signaling [85]. In this study, intraperitoneal injection of the PAR2 antagonist AZ3451 and the ERK1/2 inhibitor PD98059 significantly inhibited the *T*. *spiralis* IIL invasion of gut mucosa, as demonstrated that the worm burdens of intestinal IIL and adults were evidently reduced in the inhibitors-administrated mice. Our results also verified that AZ3451 and PD98059 up-regulated the expression of intestinal TJ proteins by inhibiting intestinal PAR2 expression and ERK1/2 phosphorylation levels, reducing the expression of pro-inflammatory cytokines, up-regulating the expression of anti-inflammatory cytokines, and ameliorating intestinal inflammation, consequently impairing the IIL invasion. In addition to trypsin secreted by *T*. *spiralis* worms, there are increased trypsin levels in the inflamed intestine, which could also induce the phosphorylation of ERK1/2 through activation of PAR2, thus regulating intestinal permeability [77, 84]. Evidently, whether the trypsin is secreted by *T*. *spiralis* or by inflamed intestine, AZ3451 could inhibit the trypsin-induced activation of PAR2, thereby reducing the phosphorylation level of ERK1/2 and up-regulating the expression of intestinal TJs proteins, consequently decreasing intestinal permeability and ameliorating intestinal inflammation. Additionally, the ERK1/2 inhibitor PD98059 had a similar effect by directly inhibiting the phosphorylation of ERK1/2. Our results indicated that rTsTryp and PAR2 agonist promoted larval invasion; whereas anti-rTsTryp antibodies, PAR2 antagonist and ERK1/2 inhibitors impeded *T*. *spiralis* larval invasion of intestinal mucosa, as demonstrated by significant reduction of enteral IIL and adult burden, these finding indicated that rTsTryp mediates larval invasion of gut epithelia via binding to PAR2 and activating the ERK1/2 pathway. However, the *in vivo* effect of rTsTryp on intestinal adult worm burdens in infected mice is necessary to be investigated in further study. These results suggested that the small molecule inhibitor (AZ3451) could be considered as an adjuvant therapeutic to impede larval invasion and relieve intestinal inflammation at the early stage of *T*. *spiralis* infection. The results further indicated that TsTryp binding to gut epithelium PAR2 activated the ERK1/2 pathway, decreased the expression of gut epithelial intercellular TJs proteins, disrupted epithelial barrier integrity, and consequently mediated larval invasion of the gut mucosa. Therefore, rTsTryp could be regarded as a potential vaccine target for blocking *T*. *spiralis* invasion and infection.

In conclusion, TsTryp was cloned and expressed in this study. TsTryp is a secretory protease that is highly expressed in the IIL invasive stage. rTsTryp binds to IECs, and the binding sites are principally localized in the IEC cytomembrane. rTsTryp has the enzymatic activity of natural trypsin to hydrolyze the substrate BAEE, but rTsTryp could not directly degrade TJs proteins. Binding of rTsTryp to the receptor PAR2 in IECs activated the ERK1/2 pathway, increased the expression of p-ERK1/2, down-regulated TJ proteins, disrupted gut epithelial barrier integrity, and caused intestinal inflammation, thereby mediated *T*. *spiralis* larval invasion of the host’s intestinal mucosa. rTsTryp promoted larval invasion, whereas anti-rTsTryp antibodies, PAR2 and ERK1/2 inhibitors impeded *in vitro* larval invasion. Two inhibitors (AZ3451 and PD98059) also impeded the invasion of the intestinal mucosa by *T*. *spiralis* and alleviated intestinal inflammation in infected mice. These results indicated that TsTryp binding to PAR2 activated the ERK1/2 pathway, decreased the expression of gut TJs proteins, disrupted epithelial integrity and barrier function, and consequently mediated larval invasion of the gut mucosa. Therefore, rTsTryp may be regarded as a potential vaccine target for blocking *T*. *spiralis* infections. The results also provide a better understanding of the mechanism of interaction between TsTryp and PAR2 in *T*. *spiralis* infection, and suggest that the PAR2 antagonist AZ3451 could be considered as an adjuvant therapeutic to impede larval invasion and relieve intestinal inflammation at the early stage of *T*. *spiralis* infection.

## Supporting information

S1 Table Special primer sequences of genes used in qPCR. (DOCX)

S1 Fig The inhibitor administration scheme and test protocol designed in this study. AZ3451 and PD98059 were intraperitoneally injected into the mice three times (days 0, 2, and 4). The mice were infected with 200 *T*. *spiralis* ML 12 h after the initiation of inhibitor administration. Ten mice from each group were euthanized to collect the IIL at 12 hours post infection (hpi), and the remaining 15 mice in each group were sacrificed at 5 dpi to collect adult intestinal worms (n = 10). Intestinal permeability was measured, intestinal tissue was collected, and the expression of PAR2, p-ERK1/2, TJs proteins, and inflammatory cytokines was determined using qPCR, western blotting (WB), and immunohistochemistry (IHC) staining at 5 dpi (n = 5).
(TIF)

S2 Fig Effects of rTsTryp on the viability of IECs and Caco-2 cells assayed by CCK-8. **A:** IEC cells were treated with different concentrations of rTsTryp for 3 h to determine cell viability. **B:** Viability of Caco-2 cells treated with different concentrations of rTsTryp for 3 h; ns: no statistical difference (*P* > 0.05).
(TIF)

S3 Fig Binding ability of rTsTryp to IEC proteins assayed by ELISA. **A:** Binding of different concentrations of IEC proteins to 2 μg/ml rTsTryp; the optimum IEC protein coating concentration was 1.5 μg/ml. **B:** Binding of 1.5 μg/ml IEC protein to different concentrations of rTsTryp. The binding of rTsTryp to IEC proteins was dose-dependent for both proteins.
(TIF)

S4 Fig Far-Western analysis of binding between rTsTryp and IEC proteins. **A:** SDS-PAGE analysis of IEC and C2C12 proteins. Lane M: protein Markers; Lane 1: IEC soluble protein; Lane 2: C2C12 soluble protein. **B:** Far-western blotting showing the binding of rTsTryp and IEC proteins. Lane M: protein markers; strips containing IEC proteins (lanes 1–9) were incubated with rTsTryp (lanes 1–3), IIL ES antigens (lanes 4–6) or TRX-tag (lanes 7–9), then probed with anti-rTsTryp serum (lanes 1, 4, and 7), infection serum (lanes 2, 5, and 8), and pre-immune serum (lanes 3, 6, and 9). Binding of rTsTryp, IIL ES antigens, and IEC proteins was identified by anti-rTsTryp serum (lanes 1 and 4) and infection serum (lanes 2 and 5), but not by pre-immune serum (lanes 3 and 6). No binding of the TRX-tag with IECs was observed using anti-rTsTryp serum (lane 7), infection serum (lane 8), or pre-immune serum (lane 9). **C:** Far-western blotting showed that rTsTryp did not bind to C2C12 protein. Lane M: protein markers; rTsTryp did not bind to C2C12 proteins when anti-rTsTryp serum (lane 1), infection serum (lane 2), or pre-immune serum (lane 3) was used.
(TIF)

S5 Fig AZ3451 and PD98059 inhibited the p-ERK1/2 expression in intestinal epithelia of *T*. *spiralis-*infected mice. Immunohistochemical results showed that the positive staining was dark brown and hematoxylin staining was blue. The expression levels of p-ERK1/2 were analyzed by gray-level analysis. *P < 0.05 compared with the saline group. ^#^*P* < 0.05 relative to AZ3451+PD98059 group.
(TIF)
