# Supplementary material for: A novel trypsin of Trichinella spiralis mediates larval invasion of gut epithelium via binding to PAR2 and activating ERK1/2 pathway
Source: PLoS Negl Trop Dis. 2024 Jan 2;18(1):e0011874. doi: 10.1371/journal.pntd.0011874 (PMC10786404; doi:10.1371/journal.pntd.0011874)
Supplement: S1 Table — (DOCX) [file pntd.0011874.s001.docx]

**Table 1. Special primer sequences of genes used in qPCR**

| Gene names | Primer | Sequence (5′-3′) | GenBank no. |
| --- | --- | --- | --- |
| ZO-1 | Forward | CGGTCCTCTGAGCCTGTAAG | XM_054378717.1 |
| (Human) | Reverse | GGATCTACATGCGACGACAA |  |
| E-cadherin | Forward | GCCTCCTGAAAAGAGAGTGGAAG | NM_131820.1 |
| (Human) | Reverse | TGGCAGTGTCTCTCCAAATCCG |  |
| Occludin | Forward | ATGGCAAAGTGAATGACAAGCGG | XM_026274194.1 |
| (Human) | Reverse | CTGTAACGAGGCTGCCTGAAGT |  |
| Claudin-1 | Forward | GTCTTTGACTCCTTGCTGAATCTG | NM_021101.5 |
| (Human) | Reverse | CACCTCATCGTCTTCCAAGCAC |  |
| GAPDH | Forward | TGTGTCCGTCGTGGATCTGA | NM_002046.7 |
| (Human) | Reverse | TTGCTGTTGAAGTCGCAGGAG |  |
| ZO-1 | Forward | TGAACGCTCTCATAAGCTTCGTAA | NM_001417372.1 |
| (Mouse) | Reverse | ACCGTACCAACCATCATTCATTG |  |
| E-cadherin | Forward | GGTCATCAGTGTGCTCACCTCT | NM_131820.1 |
| (Mouse) | Reverse | GCTGTTGTGCTCAAGCCTTCAC |  |
| Occludin | Forward | TGGCAAGCGATCATACCCAGAG | NM_001360536.1 |
| (Mouse) | Reverse | CTGCCTGAAGTCATCCACACTC |  |
| Claudin-1 | Forward | GGACTGTGGATGTCCTGCGTTT | NM_016674.4 |
| (Mouse) | Reverse | GCCAATTACCATCAAGGCTCGG |  |
| TNF-α | Forward | CCCTCACACTCAGATCATCTTCT | NM_013693.3 |
| (Mouse) | Reverse | GCTACGACGTGGGCTACAG |  |
| IL-1β | Forward | AGCTCTCCACCTCAATGGAC | NM_008361.4 |
| (Mouse) | Reverse | ATCATTGCGTGGGATCTTGA |  |
| IL-4 | Forward | TTGTCATCCTGCTCTTCTTTCT | NM_021283.2 |
| (Mouse) | Reverse | CTGTGGTGTTCTTCGTTGCT |  |
| IL-10 | Forward | CCCTTTGCTATGGTGTCCTT | NM_010548.2 |
| (Mouse) | Reverse | TGGTTTCTCTTCCCAAGACC |  |
| GAPDH | Forward | GGTTGTCTCCTGCGACTTCA | NM_001411840.1 |
| (Mouse) | Reverse | TGGTCCAGGGTTTCTTACTCC |  |
