# Supplementary figures and images for: A novel trypsin of Trichinella spiralis mediates larval invasion of gut epithelium via binding to PAR2 and activating ERK1/2 pathway
Source: PLoS Negl Trop Dis. 2024 Jan 2;18(1):e0011874. doi: 10.1371/journal.pntd.0011874 (PMC10786404; doi:10.1371/journal.pntd.0011874)

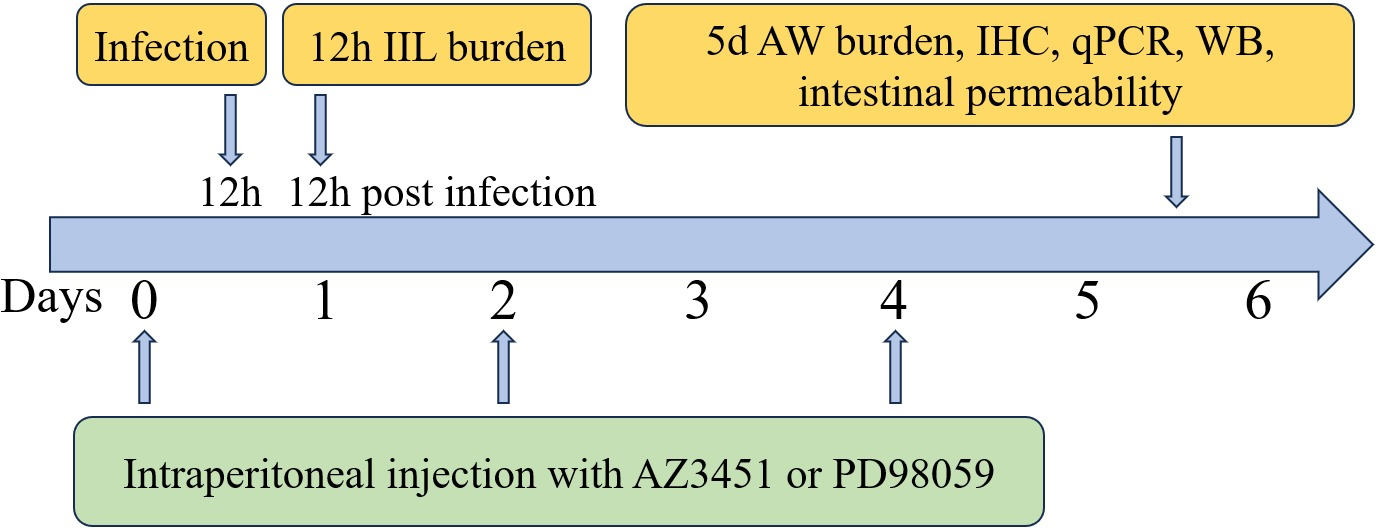

Supplement: S1 Fig — AZ3451 and PD98059 were intraperitoneally injected into the mice three times (days 0, 2, and 4). The mice were infected with 200 T. spiralis ML 12 h after the initiation of inhibitor administration. Ten mice from each group were euthanized to collect the IIL at 12 hours post infection (hpi), and the remaining 15 mice in each group were sacrificed at 5 dpi to collect adult intestinal worms (n = 10). Intestinal permeability was measured, intestinal tissue was collected, and the expression of PAR2, p-ERK1/2, TJs proteins, and inflammatory cytokines was determined using qPCR, western blotting (WB), and immunohistochemistry (IHC) staining at 5 dpi (n = 5). (TIF) [file pntd.0011874.s002.tif]

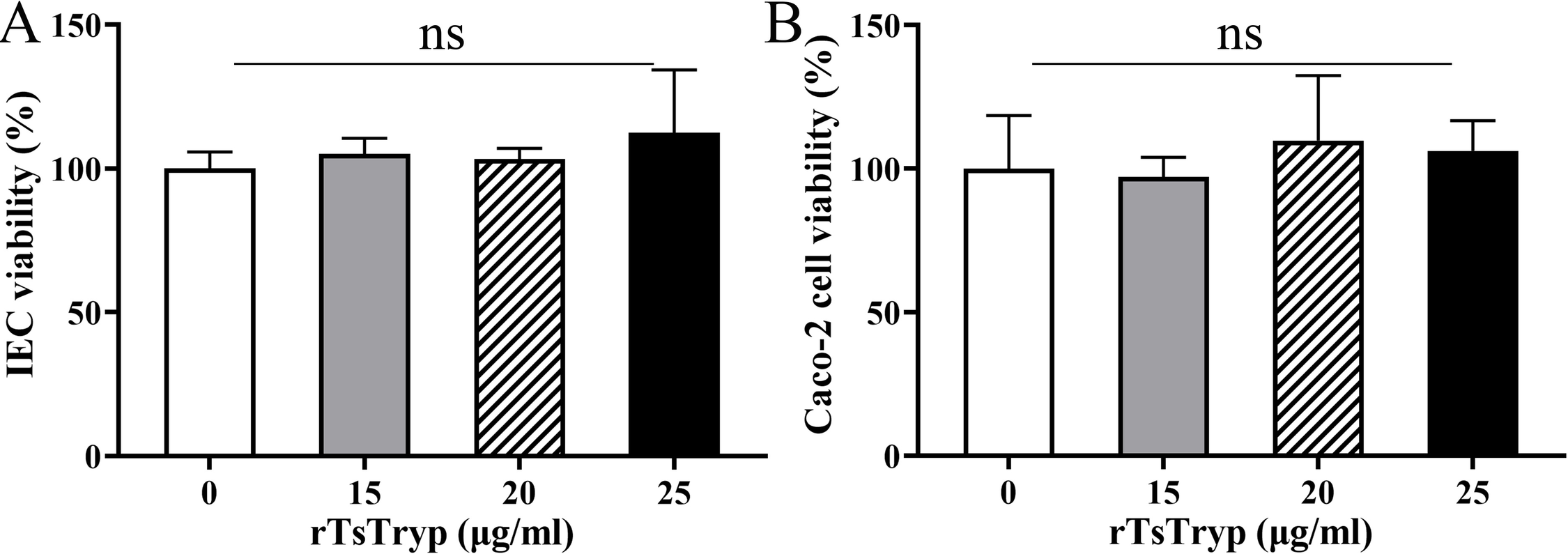

Supplement: S2 Fig — A: IEC cells were treated with different concentrations of rTsTryp for 3 h to determine cell viability. B: Viability of Caco-2 cells treated with different concentrations of rTsTryp for 3 h; ns: no statistical difference (P > 0.05). (TIF) [file pntd.0011874.s003.tif]

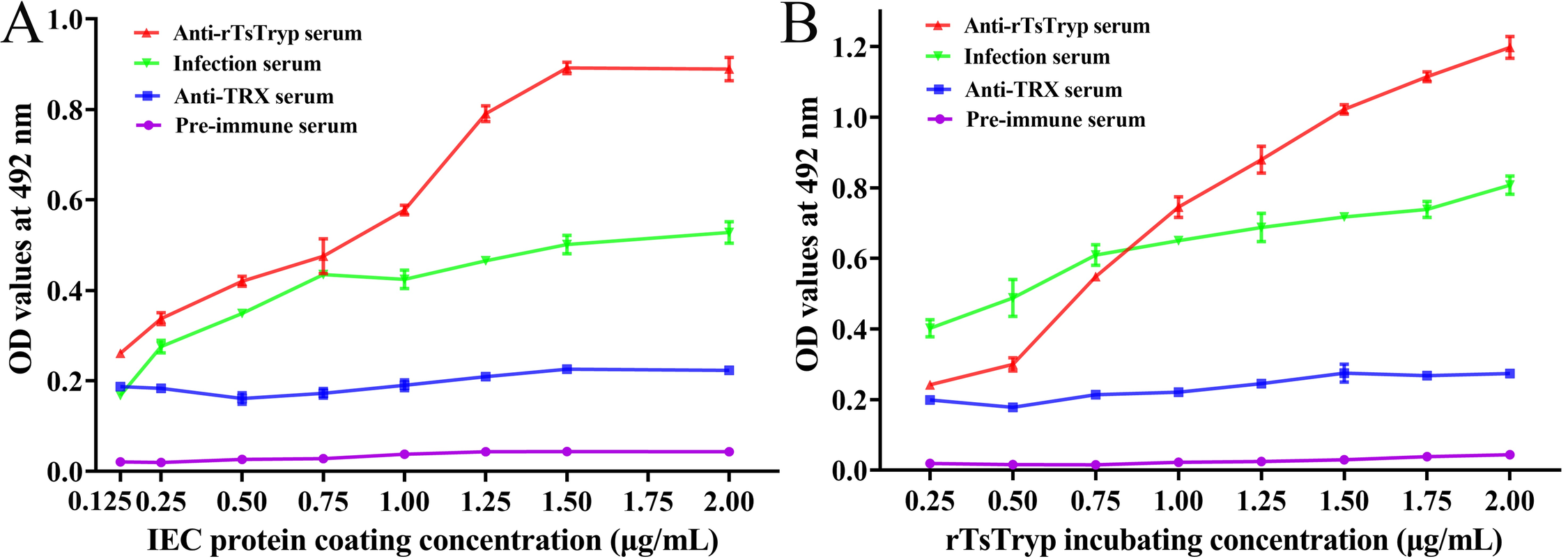

Supplement: S3 Fig — A: Binding of different concentrations of IEC proteins to 2 μg/ml rTsTryp; the optimum IEC protein coating concentration was 1.5 μg/ml. B: Binding of 1.5 μg/ml IEC protein to different concentrations of rTsTryp. The binding of rTsTryp to IEC proteins was dose-dependent for both proteins. (TIF) [file pntd.0011874.s004.tif]

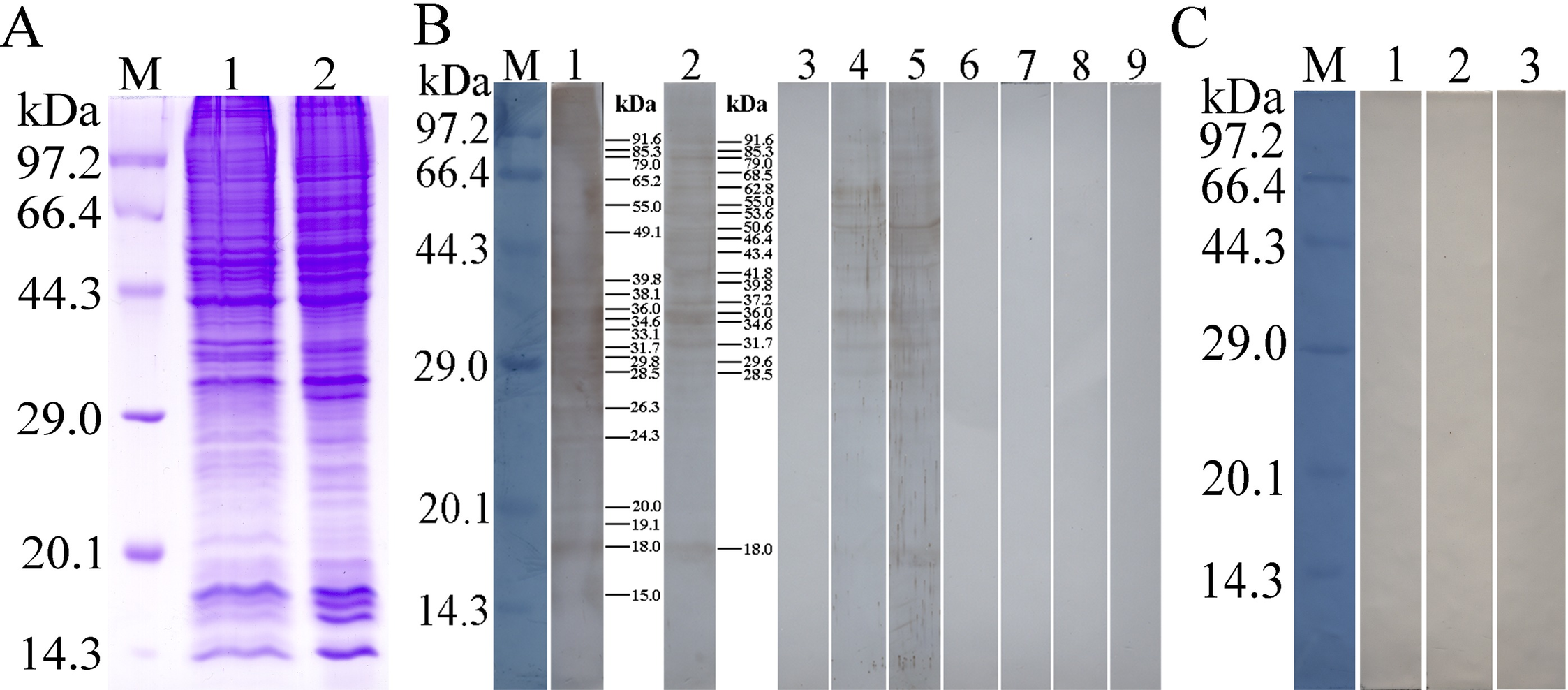

Supplement: S4 Fig — A: SDS-PAGE analysis of IEC and C2C12 proteins. Lane M: protein Markers; Lane 1: IEC soluble protein; Lane 2: C2C12 soluble protein. B: Far-western blotting showing the binding of rTsTryp and IEC proteins. Lane M: protein markers; strips containing IEC proteins (lanes 1–9) were incubated with rTsTryp (lanes 1–3), IIL ES antigens (lanes 4–6) or TRX-tag (lanes 7–9), then probed with anti-rTsTryp serum (lanes 1, 4, and 7), infection serum (lanes 2, 5, and 8), and pre-immune serum (lanes 3, 6, and 9). Binding of rTsTryp, IIL ES antigens, and IEC proteins was identified by anti-rTsTryp serum (lanes 1 and 4) and infection serum (lanes 2 and 5), but not by pre-immune serum (lanes 3 and 6). No binding of the TRX-tag with IECs was observed using anti-rTsTryp serum (lane 7), infection serum (lane 8), or pre-immune serum (lane 9). C: Far-western blotting showed that rTsTryp did not bind to C2C12 protein. Lane M: protein markers; rTsTryp did not bind to C2C12 proteins when anti-rTsTryp serum (lane 1), infection serum (lane 2), or pre-immune serum (lane 3) was used. (TIF) [file pntd.0011874.s005.tif]
